# Workers’ well-being during viral pandemics and epidemics: A scoping review

**DOI:** 10.1016/j.cpnec.2025.100286

**Published:** 2025-03-04

**Authors:** Tyler Pacheco, Simon Coulombe, Nancy L. Kocovski, Julia Carbone

**Affiliations:** a Department of Psychology, Wilfrid Laurier University, 75 University Ave W, Waterloo, Ontario, N2L 3C5, Canada; b Department of Industrial Relations, Université Laval, 2325 Rue de l'Université, Québec, Québec, G1V 0A6, Canada; c Member of the Relief Research Chair in Mental Health, Self-management, and Work, Université Laval, 2325 Rue de l'Université, Québec, Québec, G1V 0A6, Canada; d VITAM – Sustainable Health Research Centre, 2480, Chemin de la Canardière, Québec, Québec, G1J 2G1, Canada; e Relief Research Chair in Mental Health, Self-management, and Work, Université Laval, 2325 Rue de l'Université, Québec, Québec, G1V 0A6, Canada; f CERVO Brain Research Centre, 2601 Chemin de la Canardière, Québec, Québec, G1J 2G3, Canada; g Centre d’études et d'interventions en santé mentale, Université Laval, 2325 Rue de l'Université, Québec, Québec, G1V 0A6, Canada; h Centre for the Study of Democratic Citizenship, Department of Political Science, Université de Montréal, C.P. 6128 succursale Centre-Vill, Montréal, Québec, H3C 3J7, Canada; i Bloorview Research Institute, Holland Bloorview Kids Rehabilitation Hospital, 150 Kilgour Road, Toronto, Ontario, M4G 1R8, Canada

**Keywords:** Pandemic, Epidemic, Worker, Well-being, Precarious work

## Abstract

Studies have documented workers' well-being during individual pandemics and epidemics. However, there lies a need to summarize worker well-being *across* crises. Moreover, there is a scarcity of reviews exploring precarious workers' well-being during these crises. Adopting a multidisciplinary perspective via positive psychology's third wave, this scoping review examines positive and negative well-being across diverse occupational groups and situations (e.g., precarious employment) and across crises. Inspired by Ecological Systems Theory, factors at different ecological levels (self, social, workplace, pandemic) relevant to workers' well-being are reviewed. The following questions are addressed: 1) How are virus-related public health crises (i.e., epidemics, pandemics) related to workers' well-being? 2) What resilience and risk factors are associated with workers' well-being in these crises? And 2a) How is the well-being of precarious workers impacted during virus-related public health crises? Of the 2,395 potentially relevant articles published before October 23rd, 2020, 187 were retained. Overall, more research has been conducted on negative than positive well-being. Workers experienced: 1) positive well-being frequently or at moderately high levels overall during pandemics and epidemics, 2) mild to moderate negative well-being during SARS and COVID-19's beginning and high negative well-being during other crises, and 3) high work-related well-being during such crises. Factors at self- (age, gender), social- (social support), workplace- (occupation, frontline status), and pandemic-related (risk/exposure, knowing someone infected/killed by the virus, PPE access) levels were associated with workers' well-being. Although explored infrequently, precarious employment was typically associated with greater negative well-being. Practice- and policy-related recommendations are discussed.

## Highlights

• A review was needed to summarize findings on worker well-being across epi/pandemics.
• We reviewed 187 papers on resilience and risk factors associated with well-being.
• Findings show moderately high levels of positive well-being (e.g., resilience).
• Negative well-being (anxiety, depression) was found to vary across epi/pandemics.
• Precarious employment was associated with greater negative well-being.

## 1 Introduction

Viral pandemics and epidemics (defined in Table 1)^3^ have a remarkable effect on societies given that their rate of infection can impair vast regions in a short period. The ramifications of these crises surpass infection and mortality, however, as they can influence societies as a whole and the citizens that reside within them (e.g., social, economic impacts [1]). For example, simulations of the economic impact of the 2003 severe acute respiratory syndrome (SARS) outbreak suggest that the world economy suffered a cost of approximately $40 to $54 billion US dollars [2]. Similarly, the 2015 outbreak of Middle East respiratory syndrome (MERS) was related to a $2.6 billion US dollar loss in tourism in the Republic of Korea [3]. At the beginning of the COVID-19 pandemic (May 2020), 51.80% of Canadians very worried about their financial situation experienced moderate to severe anxiety [4]. More broadly, numerous studies (e.g., [[5], [6], [7], [8], [9], [10], [11], [12], [13]]) and reviews (e.g., [[14], [15]]) have documented the relationship between diverse virus-related crises (pandemics/epidemics) and workers' well-being. However, there is a need for a review that synthesizes this knowledge as the scope of what we know regarding several areas (e.g., positive well-being, the similarities and differences between virus-related crises, non-healthcare and precarious workers’ experiences) is still unclear. Such a review would 1) aid others in understanding the scope of studies that exist in the literature regarding these seemingly underexplored topics, and 2) identify what areas should be explored in later research programs [16].

Although well-being may have a direct or indirect relationship with these public health crises, diverse factors (e.g., material resources, psychological factors [17]) can be associated with one's adaptation to these external events (e.g., [18]). From Resilience Theory [19] and social determinants of mental health [20], we explore the risk and resilience factors present in one's life that can put a worker at risk of poorer well-being or protect them from negative outcomes. Consistent with Bronfenbrenner's [21] Ecological Systems Theory, factors can be proximal (i.e., closer to workers; e.g., age, gender), or distal (i.e., farther away from workers; e.g., risk of infection, social distancing). Adopting this perspective, our review investigates the factors researchers have explored during pandemics and epidemics.

There seem to be characteristics that are associated with well-being across different crises. For example, Brooks et al. [22] identified several social and occupational factors (e.g., working in high-risk environments, specialized training) that were related to healthcare workers' mental health during the 2002 SARS outbreak. Comparably, Luo et al. [23] described multiple factors (e.g., contact with infected patients, family support) that were related to medical staff's well-being at COVID-19's onset. Given the diversity of factors in the literature, a review is needed to summarize all the studies conducted on such factors and their relationship with well-being.

### 1.1 Holistically understanding well-being

A traditional well-being perspective explores a pathogenic (i.e., disease-oriented) approach, which views health as the absence of disability, disease, and premature death [24]. This perspective may refer to well-being as the presence or severity of distress and anxiety, for example. A second approach aligned with positive psychology, labeled as salutogenic, focuses on positive human capacities and functioning [24]. This salutogenic approach may refer to well-being as the presence of flourishing and resilience, for example.

Beyond well-being experienced in general in one's life, well-being can also be examined from a domain-specific perspective [25]. At work, negative well-being may manifest as low energy or burnout and absenteeism or presenteeism [26]. As described by Jeong et al. [27] (p. 1), “Absenteeism and presenteeism refer to loss in productivity related to sick leave and illness, respectively.” Positive well-being at work may include job satisfaction [28] or job performance [29]. A review by Lomas et al. [28], reminiscent of the Two Continua Model [30,31], showed how the pathogenic and salutogenic continua can be unified to provide a holistic portrait of well-being that considers work-related and general life well-being. In their review on the effects of mindfulness-based interventions, irrespective of the domain, mindfulness was found to have moderate effects on negative well-being indicators (e.g., burnout, stress, anxiety) and small to moderate effects on positive well-being indicators (e.g., job performance, positive well-being) [28]. Inspired by this approach, we explored how pandemics and epidemics may be differently associated with workers' negative and positive psychological well-being at work and in their general lives.

## 2 Limitations of past scoping reviews

Reviews published to date predominantly focused on workers' well-being: 1) from a disease-oriented perspective (e.g., [15,32]), 2) in a singular pandemic or epidemic (e.g., [22,23]), and/or 3) within a specific occupational group, such as health care workers (e.g., [14,22]). Such focus on the disease-oriented perspective suggests a general lack of exploration of positive well-being outcomes during pandemics and epidemics (e.g., COVID-19; also see: [33]). Although aiding in understanding a particular set of circumstances, reviews that unify information regarding a diverse range of workers’ positive and negative well-being in the context of several virus-related crises would represent an unparalleled resource.

Another limitation of past reviews is the lack of focus on precarious employment. Precarious employment has three dimensions: employment insecurity, income inadequacy, and lack of protection and rights [34]. These three dimensions have a deleterious relationship with well-being in non-pandemic (e.g., [35]) and pandemic-related (e.g., onset of COVID-19; [36]) circumstances. Moreover, precarious employment has a negative influence on multiple other risk factors (including social determinants of health, e.g., [37]). With how harmful precarious employment can be, attention is given in the present scoping review to see whether, and, if so, how, dimensions of precarious employment are associated with well-being across different virus-related crises.

A synthesized review would have several implications for diverse stakeholders. Firstly, a review is essential for mental health practitioners and workplace stakeholders (e.g., employers) as they can help develop primary prevention interventions during future public health crises by 1) reducing job-related stressors caused by crises at the source by modifying the job itself or the work environment [38], and 2) helping to find ways to enhance workers' strengths and capabilities [39]. Second, a synthesized review will provide valuable information to positive psychologists and happiness studies scholars. As noted by Wissing [40], the current third wave of positive psychology is a frontier for the multidisciplinary study of individuals in their social systems. This multidisciplinary perspective becomes particularly relevant when better understanding complex issues that affect well-being [40], such as pandemics and epidemics. It is to our knowledge that, although many have taken a perspective rooted in positive psychology to better understand how pandemics and epidemics affect worker well-being, many have not drawn on third wave's multidisciplinary nature to further our understanding of these phenomena. Theories within positive psychology (Resilience Theory [19]; The Two Continua model [30,31]), industrial-organizational psychology (precarious work [34]), public health (Social Determinants of Mental Health [20]), and developmental and community psychology (Ecological Systems Theory [21]) are drawn upon in this review to better understand workers' well-being during such crises. It is thought that using the third wave of positive psychology to marry these complementary perspectives in this review will provide a practical example of how researchers can use the third wave as a tool to holistically understand how workers are impacted during pandemics and epidemics.

### 2.1 The present scoping review

The present scoping review explored the following research questions:

1. How are virus-related public health crises (i.e., epidemics, pandemics) related to workers' well-being?
2. What resilience and risk factors were found to be associated with workers' well-being in the context of virus-related public health crises?
  2a. How is the well-being of precarious workers impacted during virus-related public health crises?

## 3 Methods

Conducting a scoping review allows researchers to assess the size and scope of the publicly available literature [41]. Scoping reviews (versus other review types) ensures that the nature and extent of the research could be reported on while also providing a narrative summary of how workers' well-being is impacted by such crises. An adapted version of Arksey and O'Malley's [42] review protocol was used, which is described hereafter. Tricco et al.’s [43] Preferred Reporting Items for Systematic Reviews and Meta-Analyses extension for Scoping Reviews (PRISMA-ScR) checklist was used to ensure essential items were reported on.

### 3.1 Stage one: identifying the research questions

The research questions of the scoping review were developed with the aid of the PICO strategy, which takes into consideration the person(s) (P), intervention (I), context (C), and outcomes (O) of interest [44]. The “persons” of interest are all forms of paid workers. Like other non-experimental reviews (e.g., [45,46]), the intervention, or the phenomenon of interest in the context of non-experimental studies as it is the case here, is employment in the context of pandemics and epidemics. More specifically, regarding the context, it refers to the beginning of COVID-19, as well as any other virus-related crises worldwide (e.g., MERS, SARS, Ebola virus disease [EVD]). The term “COVID-19 [pandemic]” is used hereafter for brevity to reflect the beginning of the pandemic. Lastly, workers' well-being is explored as the main outcome of interest. Inspired by Lomas et al.’ [28] holistic well-being approach, the scoping review explored dimensions of positive (e.g., flourishing, resilience) and negative well-being (e.g., anxiety, depression) in workers’ general lives as well as dimensions of work well-being (e.g., willingness to work, burnout, job satisfaction).

### 3.2 Stage two: identifying relevant studies

The authors established: 1) the databases used to search for relevant articles, 2) the search strategy used to collect potentially relevant articles from the databases, and 3) the eligibility criteria that determined the articles that should be retained for the scoping review.

#### 3.2.1 Databases

A psychology subject librarian at Wilfrid Laurier University was consulted to determine the databases we should use to answer our research questions. All three recommended databases were used in our review. First, MEDLINE was used to explore a life science and biomedical perspective. Second, PsycINFO provided articles that examined any link between these crises and the psyche. Last, CINAHL was used to gain insight into other relevant articles related to nursing, applied health, biomedicine, and healthcare. Respectively, 1,315, 653, and 553 articles were exported from these databases. Once SciWheel (a reference management website) removed duplicate files, there were 2,395 articles that entered the study selection phase.

#### 3.2.2 Search strategy

As three different databases using different search query syntax were used, a different search strategy had to be used for each database. This included terms such as: “worker,” “pandemic,” “epidemic,” “well-being,” and “performance.” These terms were searched in the databases' thesauri to determine relevant terms (i.e., machine language) that are used by each database to categorize all the articles. The laypersons and machine language terms were combined and fed into the databases to search for relevant information in the titles, abstracts, and keywords of all the articles in the databases. Appendix A contains the terms used in the searches. Settings were also set only to include empirical articles written in English or French. Search results, including the article's information (e.g., titles, abstract), were exported as .RIS files and imported to SciWheel for stage three of the scoping review.

#### 3.2.3 Eligibility criteria

Eligible articles were those: written in English or French (the two languages of Canada where the authors are working and the only languages the research team could read), empirical, observational, peer-reviewed (or a dissertation/thesis), contextualized within a pandemic or epidemic, exploring one or more constructs related to positive and/or negative well-being, that have a quantitative component (solely quantitative or a mixed methods study), and with a (sub)sample that is at least 50% workers. Although most reviews implement a specific start and end date when the articles had to be published, the presented scoping review did not establish a start date to ensure that empirical research regarding workers' well-being during all relevant past public health crises was captured. All articles had to be published by October 22nd, 2020, as this was when the study selection phase began. As COVID-19-related research was continuously being published, the selection phase was intentionally delayed allowing more time for peer-reviewed articles to be published. However, with no end to the pandemic in sight (thus meaning that COVID-19-related research was going to be continuously published), a decision was made to begin the study selection phase (see Limitations section for more information on the impact of this decision). As the present scoping review aims to explore workers’ well-being in the context of viral outbreaks, articles that solely explore chronic (e.g., HIV/AIDS), seasonal (e.g., seasonal flu), or hypothetical/future public health crises were excluded from this review. Regarding the decision to exclude research on HIV/AIDS epidemics, the pathogen is different from other respiratory/communicable pathogens. As noted by Haseltine [47], the tracing and quarantining of COVID-19 has made it possible to contain the pathogen, thus making regions mostly pathogen-free. This is arguably the case with several other pathogens (e.g., H1N1, SARS, MERS, EVD) responsible for epidemics and pandemics. However, the HIV/AIDS epidemic has not been containable in the last 35 years despite the implementation of public health measures and biomedical research. The eligibility criteria served as verification questions during the two article retention phases (see stage three).

### 3.3 Stage three: study selection

The first author and a trained research assistant (RA) completed two screening phases to determine which articles should be retained. The first phase of excluding irrelevant articles involved reviewing the articles' title and abstract. Articles with abstracts containing insufficient detail entered a second phase where the articles’ full text was reviewed.

#### 3.3.1 Phase one

For each paper, six questions (Table 1) were answered in a successive fashion to help guide the evaluation process. After reading the title and abstract, the two reviewers individually answered “Yes,” “No,” or “Maybe” to each question. As soon as one answer was identified to be “No,” the article was excluded, and the remaining questions were not answered. The reviewers and the second author met weekly to review any discrepancies in the review process. The second author met with the two reviewers and acted as a third party when the two reviewers reached a different decision during the evaluation process. When discrepancies occurred, the three collectively determined whether to retain or exclude articles, or, alternatively, put them through a secondary review process (see phase two). This included cases where one reviewer answered “Yes” (indicating article retention) and the other “Maybe” (indicating possible retention, but not enough information is provided) to one or multiple question(s). The inter-rater reliability between the two reviewers during this stage was moderate (kappa = 0.69 [48]). At the end of phase one, articles that were not excluded either entered the second phase of article retention or went to stage four for data extraction. Those in the former were articles with a “Maybe” (but no “No”) on one or more questions. This indicated that the abstract and title did not provide sufficient evidence of whether the article fits the eligibility criteria. Those with “Yes” for every question went to data extraction as the information was sufficient in determining that the article fit the review’s aim. Of the 2,395 articles in phase one, 2,132 were excluded, 99 entered phase two of study selection, and 164 were retained and entered data extraction.

**Table 1:** Questions used to determine article eligibility.

Questions	Answer
*Is the study focused on a pandemic or epidemic?* A **pandemic** is “an epidemic occurring worldwide, or over a very wide area, crossing international boundaries and usually affecting a large number of people” ([49], p. 131). An **epidemic** is “the occurrence in a community or region of cases of an illness, specific health-related behaviour, or other health-related events clearly in excess of normal expectancy. The community or region and the period in which the cases occur are specified precisely” [50]. This includes (but is not limited to): Cholera, Ebola virus disease, Influenza (pandemic, zoonotic), MERS-CoV, Novel coronavirus (2019-nCoV), Plague, Tuberculosis, and Yellow fever. If you are unsure, search for the disease online to see if it is considered a pandemic or an epidemic. Chronic or cyclic pandemics and epidemics that can be automatically removed include (but are not limited to): seasonal flu, obesity, cancer, and HIV/AIDS.	*Yes/No/Maybe*
*Is the study focused on the impact of the crisis on human well-being as measured by one or more of the following outcome variables: well-being, wellness, mental health, anxiety, burnout, depression, distress, fear, stress, performance, productivity, job satisfaction, morale, self-compassion, character strength, resilience, absenteeism, presenteeism, social isolation, and relationship well-being?* If outcome variables do not align directly with those mentioned above, but the author(s) of the reviewed paper believe(s) it could be part(s) of well-being, please note uncertainty and what specific outcomes are mentioned.	*Yes/No/Maybe*
*Is the study empirical* (e.g., not a review or a theory paper)?	*Yes/No/Maybe*
*Does the study use quantitative or mixed methods?*	*Yes/No/Maybe*
*Is the study observational in nature?* “[…] in an observational study, the investigator does not intervene and rather simply “observes” and assesses the strength of the relationship between an exposure and disease variable. Three types of observational studies include cohort studies, case-control studies, and cross-sectional studies” ([51], p. 2). This excludes intervention research and program evaluations (i.e., discussing a program and/or its efficiency).	*Yes/No/Maybe*
*Are at least 50.00% of the study's sample (or of its reported subsamples) workers*? Workers include those who were/are employed workers during the crisis in question or had a job before the crisis but had been laid off temporarily or indefinitely.	*Yes/No/Maybe*

#### 3.3.2 Phase two

**Fig. 1:**
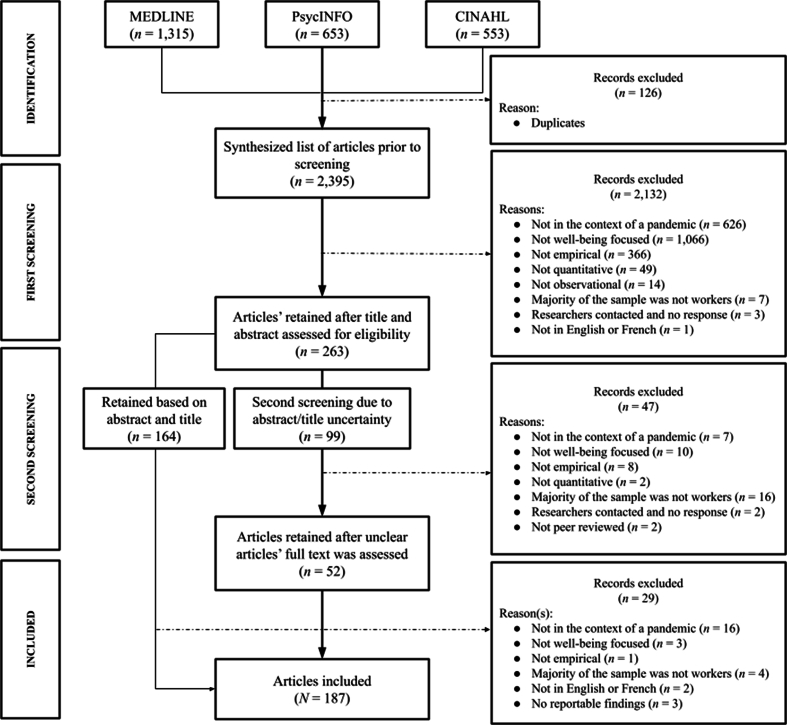
Summary of the article retention process.

Phase two consisted of reviewing the full text of the 99 articles that had at least one “Maybe” across the questions in Table 1. Sections of the papers relevant to the questions the reviewers were unsure about were reviewed and given a “Yes” or “No” if the content of the article's full text (and its supplementary materials) was sufficient in determining whether articles should be retained or not. A meeting between the two reviewers and the second author was held after this phase to resolve discrepancies. These meetings were also used to discuss unavailable articles. If the article was unavailable, the first author contacted the corresponding author to obtain the manuscripts. If the author did not respond, their article was excluded. The number of articles that were excluded for these reasons is in Fig. 1. Articles that did not have sufficient information to demonstrate retention were excluded; those that were retained entered stage four for data extraction. Of the 99 articles that entered phase two, 52 were added to the final list of retained articles. The other 47 articles were excluded.

### 3.4 Stage four: charting the data

Articles that appeared to fit the scope of the review (i.e., had “Yes” across all the screening questions) were sent to data extraction and were screened again to ensure eligibility. There were cases in which the information presented within the abstract fit the scope of the review, but further examination depicted otherwise. For example, some articles mentioned influenza pandemic in the abstract, but the full paper showed that they referred to seasonal influenza, which did not fit the aim of our review. Articles such as these were excluded at this stage. Two additional RAs were trained on the aims of the scoping review and brought forward any articles they did not believe should be retained. The first and second authors then deliberated whether the articles should be retained or excluded from the review. Twenty-nine articles were excluded; 135 were included in the final list of retained articles. When considering the 52 articles retained in phase two, overall, 187 articles were retained for the scoping review (see supplementary material for list of retained articles). A summary of the process until the end of stage four is in Fig. 1. The two RAs were trained to extract relevant information (e.g., sample characteristics, objectives, findings) from the retained articles. For training and consistency purposes, 20% of the retained articles were extracted by both RAs. The first author reviewed the extracted data to ensure that the RAs extracted and organized information consistently. Both RAs extracted information from the same articles until they did so consistently.

### 3.5 Stage five: summarising and reporting findings

Our findings are written into two subsections within the article's results section to address our research questions. The first research question (RQ1), which aims to explore workers' well-being during virus-related crises, is divided into three categories: positive well-being, negative well-being, and work-related well-being. Given the number of publications (*n* = 139) including negative well-being measures, subsections were developed to help compare the COVID-19 pandemic to past pandemics and epidemics on negative well-being.

The descriptive statistics regarding workers’ negative well-being in their general lives (hereafter called general well-being) and work-related well-being were explored to investigate whether or not poor levels of well-being were experienced during pandemics and epidemics. If at least 50.00% of the sample experienced lower symptom severity (e.g., normal to moderate levels) on a negative indicator measured in a study, the sample was considered to have “better well-being” on that well-being indicator. If *more* than 50.00% experienced higher symptom severity (e.g., severe levels), the sample was considered to have “worse well-being.” As negative well-being is often classified by symptom severity (e.g., normal, mild, moderate, severe), exploring the proportion of workers that fit into these categories allowed us to compare findings across articles irrespective of the well-being indicator and Likert scale used. The mean score was examined in cases where it was reported instead of a percentage. If the mean score on a negative well-being indicator was lower or equal to the midpoint (in the direction of expressing better well-being), the sample was considered to have “better well-being.” The sample was considered to have “worse well-being” when the mean score was higher than the midpoint (in the direction of expressing worse well-being). Irrespective of the proportion of workers experiencing higher symptom severity or reported, mean scores were considered indicative of “worse well-being” if the sample met a clinical cut-off score of a measure used to investigate the presence of mental health concerns (e.g., depression, anxiety).

A slightly different method of analyzing the data was used for positive well-being given the limited number of articles that explore this well-being dimension. Rather than using a strict analysis of descriptive statistics, the first and second author examined the texts to determine if any overarching pattern was present in the literature. When reviewing the articles, there were instances in which the findings were unreportable. For example, some articles referred to mean scores of different demographic groups (e.g., medical vs. non-medical workers). These findings overlap with our second objective, and, thus, were not included when identifying general well-being trends (RQ1).

Regarding the second research question (RQ2), risk and resilience factors were explored. As numerous factors were explored in the literature, the three most frequently tested risk and resilience factors found at the different levels (self, social, workplace, pandemic) were reported on.

## 4 Results

### 4.1 Study descriptives

As shown in Table 2, most of the retained articles were published in 2020 (74.33%), contextualized in Asia (65.78%), and focused on the COVID-19 pandemic (74.33%). Most (90.37%) used a cross-sectional design and did not explore a construct related to precarious employment (89.84%). Healthcare workers were the main occupational group of interest (84.49%). Overall, 13.90% of the articles explored at least one construct related to positive well-being; 92.51% explored at least one construct related to negative well-being. Additionally, 29.95% of the articles explore at least one construct regarding well-being at work.

**Table 2:** Summary of retained studies’ characteristics (*N* = 187).

**Aspects**	**Frequency (*n*)**	**Percentage (%)**
**Article descriptives**		

*Year of study (publication year)*		
2000 to 2009	32	17.11
2010 to 2019	16	8.56
2020	139	74.33
*Region (continent) of study*^a^		
Africa	4	2.14
Asia	123	65.78
Australia	2	1.07
Europe	28	14.97
North America	27	14.44
South America	4	2.14
*Pandemic(s) or epidemic(s) of interest*		
COVID-19	139	74.33
EVD	2	1.07
H1N1	4	2.14
MERS	6	3.21
SARS	33	17.65
Other	3	1.60
*Study design*		
Cross-sectional	170	90.37
Longitudinal	15	8.02
Mixed of the above	2	1.07
*Explored precarious employment*		
Yes	19	10.16
No	168	89.84
*Occupational group(s) explored*		
Education	7	3.74
First Responder	6	3.21
Food	2	1.07
Healthcare	158	84.49
Manufacturing	1	0.53
Social services	4	2.14
Services	12	6.42
Unspecified	21	11.23
Other	29	15.51

**Explored dependent variables**		

*Positive well-being*	26	13.90
*Negative well-being*	173	92.51
Anxiety	113	60.43
Depression	65	34.76
Distress	34	18.18
Post-traumatic stress	35	18.72
Social isolation	10	5.35
Stress	58	31.02
Worry	21	11.23
Other negative well-being	25	13.37
*Work-related well-being*	56	29.95
Absenteeism/presenteeism	1	0.53
Burnout	25	13.37
Job satisfaction	11	5.88
Morale	1	0.53
Performance	6	3.21
Willingness to work	20	10.70
Other work-related	8	4.28
*Unspecified well-being*	11	5.88

*Note.* COVID-19 = COVID-19 pandemic, EVD = Ebola virus disease, H1N1 = Swine flu, MERS = Middle East respiratory syndrome, SARS = Severe acute respiratory syndrome. Constructs with a frequency of zero (0) are removed from the table for brevity. ^a^Some countries included in this review occupy two continents. These countries (Egypt, Russia, Turkey) are added to the tally of the continent their capital city is situated. Georgia is counted with other countries in Europe.

### 4.2 Positive well-being

The articles that explored positive well-being varied in the specific constructs explored. Examples include hardiness (i.e., resilience [6,52,53]), social connectedness and activities [54,55], and positive affect [56]. Generalized across pandemics and epidemics, we identified one overarching trend based on cross-sectional findings.

General (i.e., non-work-related) positive well-being was experienced moderately to highly *or* experienced quite frequently by workers in most studies that included positive well-being. Some articles that report mean scores to represent general positive well-being show that workers' mean scores are *at least* at the midpoint of the Likert scale(s) used (e.g., [6,8]). However, researchers that explored general positive well-being primarily did so by measuring the proportion of workers experiencing different forms of this well-being (e.g., [9,[11], [12], [13]]). These articles show the presence of some levels of general positive well-being during COVID-19 as well as during previous pandemics and epidemics. For example, a considerable proportion of healthcare workers in Pakistan experienced motivation (33.30%) and hope (56.70%) during COVID-19 [57]. Other researchers found similar results during COVID-19. Zhang et al. [13] showed that several healthcare workers in China reported being calm (39.10 %) and confident (31.10 %). McAlonan et al. [9] found that 35.80% of healthcare workers in Hong Kong reported feeling bravery and unity with others during SARS. McAlonan and colleagues' [9] findings illustrate another trend amongst the retained articles’ findings: most of the sample experienced positive well-being, or, experienced it more than negative well-being. Most healthcare workers in Taiwan reported a positive attitude (65.00–69.00%) after finding out about their assignment to a team of SARS workers [58]. Similarly, McAlonan et al. [9] found that workers picked positive emotional reactions (35.80%) more than negative reactions (14.60%) when learning about their mission of taking care of SARS patients. Most workers sampled during the 2009 influenza A pandemic [59] and COVID-19 [[11], [12], [13]] show a similar pattern in that they report general positive well-being.

Limited research explored the longitudinal variation of positive well-being during pandemics and epidemics. Only six of the 26 articles (23.08%) that explored positive well-being were longitudinal. However, only four of the six longitudinal studies explored a difference in positive well-being between more than one time point [[6], [54], [60], [61], [62]]. All four studies compared workers' positive well-being before and during COVID-19, which limits the ability to cross-compare workers' experiences in different pandemics and epidemics. Across the four studies, however, the findings suggest that positive well-being decreased after the onset of a pandemic or epidemic. For example, participants’ moods decreased from 7.04 to 6.80 (Likert scale: 1–10) between 2019 and 2020 [12,60].

### 4.3 Negative well-being

Recall that negative well-being is divided into two sub-sections: past pandemics and COVID-19. Here, we first discuss workers’ negative well-being during past crises (e.g., SARS, MERS). We then discuss the same during COVID-19.

#### 4.3.1 Past pandemics and epidemics

Of the 48 articles contextualized within past pandemics and epidemics (before COVID-19), 42 (87.50%) explored at least one indicator of general negative well-being. We identified one overarching trend based on cross-sectional findings.

Irrespective of the explored pandemic and epidemic, findings on whether most of the workers sampled were experiencing worse well-being are mixed. Nineteen of the 38 retained articles (50.00%) that presented reportable findings showed that most of the samples were doing well (i.e., not experiencing the mental health concern[s] and/or experiencing the concern[s] mildly to moderately). However, 36.84% showed that most of the samples were not doing well. The other 13.16% of the articles were mixed (i.e., low on some negative well-being indicators and high on others). Negative well-being was experienced in various levels by workers depending on the crisis. Regarding SARS crises, 60.71% of the retained articles showed that most workers were doing well (i.e., relatively low levels or percentage of mental health concerns). For example, a minority of healthcare workers experienced psychiatric symptoms (27.00%) and post-traumatic stress disorder (20.00%, [63]). These results contrasted the 25.00% of articles that show that workers are not doing well (i.e., experiencing mental health concerns and/or experiencing them more than moderately) during SARS crises. The other 14.29% of the articles were mixed. Regarding other pandemics and epidemics (i.e., EVD, H1N1, H7N9, MERS), 70.00% (*n* = 7) of the retained articles showed that most of the sample was *not* doing well. For example, most sampled healthcare workers in Alberta, Canada, experienced worry regarding contracting H1N1 due to their work (64.00%) and infecting their families (77.00%, [7]). These findings contrast with 20.00% (*n* = 2) of the retained articles that showed most of the workers sampled were doing well (i.e., relatively low levels or percentage of mental health concerns) during the non-SARS-related crises. The other 10.00% of the articles were mixed.

No clear trend was present when investigating workers’ negative well-being longitudinally. Of the articles that explored negative well-being during past crises, only six articles had reportable findings [[64], [65], [66], [67], [68], [69]]. One of the six articles showed some improvement regarding mental health concerns, three showed mixed results, and one reported no difference over time. The temporal framing of these articles also varied.

#### 4.3.2 The COVID-19 pandemic

Of the 139 articles contextualized during COVID-19's onset, 130 (93.53%) explored at least one indicator of general negative well-being. Across those that explored negative well-being during COVID-19 cross-sectionally, we identified one overarching trend.

Findings from 62 (47.69%) of the retained articles showed that most workers experienced low negative well-being. A large proportion of these studies were contextualized in North America (e.g., [54,55]), (South East) Asia (e.g., [12,60,70]) and the Middle East (e.g., [71,72]). Thus, lower negative well-being appears to be a worldwide occurrence. For example, most healthcare workers in China had what they called “normal” levels (i.e., non-pathological) of anxiety (79.40%) and depression (71.30%, [70]). Similarly, a small proportion of workers in the United Arab Emirates experienced anxiety- (23.00%) and depression-related (20.00%) symptoms [73]. Although this common trend is found, a few studies show otherwise. Some researchers (20.77%) show that most workers are experiencing high negative well-being. The remaining 21.54% report that most workers are experiencing low negative well-being on some negative well-being indicators while experiencing high negative well-being on others. Articles showing that workers are experiencing high negative well-being or mixed findings are also contextualized in North America, (South East) Asia, and the Middle East.

No trends are present when investigating workers' negative well-being longitudinally at COVID-19's onset. Of the 130 that explored negative well-being during COVID-19, 13 had a longitudinal component. However, only nine [5,6,54,60,62,[74], [75], [76], [77]] had reportable findings. The temporal framing of these studies also differed. Some explored the difference before and after the onset of COVID-19. Others explored negative well-being over time during the pandemic. Three of the nine articles showed an improvement on workers’ negative well-being indicators over time, five showed negative well-being getting worse over time, and one showed no difference over time.

### 4.4 Work-related well-being

The retained articles also provided evidence suggesting that domain-specific well-being associated with one's work may have a different relationship with pandemics and epidemics when compared to general negative and positive well-being. Generalized across the pandemics and epidemics explored, we identified two overarching trends based on cross-sectional and longitudinal findings.

First, most workers reported normal or low maladaptive scores (i.e., mild, moderate) on well-being at work outcomes. Of the 43 articles with reportable findings, 25 (58.14%) contained consistent findings supporting this trend. For example, 83.40% of nurses were willing to practice in Hubei during COVID-19 [78]. Similar results were found for many other workplace well-being indicators, including absenteeism (e.g., [79]) and job engagement (e.g., [80]). However, there was nuance when exploring the differences between individual crises. Articles contextualized during SARS outbreaks reported the highest proportion (80.00%) of consistent findings showing that workers reported normal or low maladaptive scores on work-related well-being indicators. This is followed by articles contextualized during other less frequently explored pandemics and epidemics (e.g., MERS, H1N1; 62.50%) and COVID-19 (53.33%).

Second, work-related well-being typically gets worse after COVID-19's onset. Of the seven articles that included reportable longitudinal findings [62,75,[80], [81], [82], [83], [84]], four (57.14%) showed this trend. The temporal framing of these articles also varied greatly, and various indicators were reported (e.g., burnout, performance). For example, emergency medicine physicians experienced more burnout after COVID-19's onset than before (median scores: 4 and 3, respectively; [82]). Although work-related well-being does not seem to be greatly impacted negatively *or* seems to be only impacted mildly to moderately when explored at one timepoint, workers tend to experience more work-related suffering over time.

### 4.5 Risk and resilience factors

The three most frequently explored factors related to the self, social, work, and pandemics/epidemics are reported (see Table 3). For each factor, there are several publications that show whether there is a relationship between the factor and well-being, but some findings are conflicting or miscellaneous. Conflicting results are when multiple statistical tests examine factors’ relationship with well-being, and the pattern of the results is different. Miscellaneous findings are patterns of results that are curvilinear or when a tested interaction cannot be disentangled. As some findings are conflicting or miscellaneous, we consider the articles that generally report a relationship but only the articles that use significance testing to determine which groups have better well-being.

**Table 3:** Summary of findings from most frequently explored risk and resilience factors.

**Self-related Factors**		**Social-related Factors**		**Workplace-related Factors**		**Pandemic-related Factors**	
***Factors***	***n***	***Factors***	***n***	***Factors***	***n***	***Factors***	***n***
*Gender*	89	*Marriage*	51	*Occupation*	71	*Risk or exposure*	22
Not related to well-being	24	Not related to well-being	30	Not related to well-being	18	Not related to well-being	0
Related to well-being	65	Related to well-being	21	Related to well-being	53	Related to well-being	22
Men higher than Women	54	Married higher than not married	7	See Table 4 for breakdown		High higher than low risk/exposure	0
Women higher than men	8	Not married higher than married	6			Low higher than high risk/exposure	15
Conflicting findings	1	Conflicting findings	7			Conflicting findings	4
Miscellaneous findings	2	Miscellaneous findings	1			Miscellaneous findings	2
No significance test	0	No significance test	0			No significance test	1

*Age*	76	*Parenthood*	19	*Seniority or experience*	16	*Known infection or death in close circle*	16
Not related to well-being	27	Not related to well-being	12	Not related to well-being	6	Not related to well-being	3
Related to well-being	49	Related to well-being	7	Related to well-being	10	Related to well-being	13
Younger higher than older	3	Being a parent higher than not	0	Less higher than more	3	No infection higher than infection	9
Older higher than younger	33	Not a parent higher than are one	3	More higher than less	4	Infection higher than no infection	0
Conflicting findings	8	Conflicting findings	2	Conflicting findings	1	Conflicting findings	2
Miscellaneous findings	3	Miscellaneous findings	2	Miscellaneous findings	2	Miscellaneous findings	1
No significance test	2	No significance test	0	No significance test	0	No significance test	1

*Education*	41	*Social support*	9	*Working frontline*	14	*PPE access*	14
Not related to well-being	26	Not related to well-being	1	Not related to well-being	0	Not related to well-being	2
Related to well-being	15	Related to well-being	8	Related to well-being	14	Related to well-being	12
Less higher than more	4	Supported higher than not	3	Frontline higher than other	1	Limited higher than adequate access	1
More higher than less	8	Not supported higher than are	0	Other higher than frontline	12	Adequate higher than limited access	5
Conflicting findings	1	Conflicting findings	1	Conflicting findings	1	Conflicting findings	0
Miscellaneous findings	2	Miscellaneous findings	0	Miscellaneous findings	0	Miscellaneous findings	1
No significance test	0	No significance test	4	No significance test	0	No significance test	5

*Note*. Conflicting findings are when multiple statistical tests are used to explore the relationship between the factor and one or more well-being constructs, and the pattern of the results is not the same. Miscellaneous findings are patterns of results that are curvilinear or when there is an interaction tested that cannot be disentangled. No significance test refers to findings that did not use a statistical test to explore the relationship between the factor and well-being or did not report statistical tests allowing to identify which specific groups were different from one another. *N* = frequency.

#### 4.5.1 Self-related factors

Self-related factors are the most proximal category. Self-related factors are either representative of a) the workers themselves (e.g., age) or b) characteristics that workers have received/acquired throughout their life (e.g., education). Of the 187 retained articles, 128 (68.45%) explored how self-related factors were related to well-being. Gender (*n* = 89), age (*n* = 76), and education (*n* = 41) were the most explored factors. Men typically had better well-being than women, as demonstrated by 54 of the 65 articles statistically investigating the relationship between gender and well-being. However, the relationship was mainly found during COVID-19. For example, female (vs. male) healthcare workers in India were more likely to experience anxiety-related symptoms during COVID-19 [85]. The finding that women had worse well-being than men was replicated in other countries during COVID-19, such as Israel [10], China [86,87], and Egypt [88]. For SARS, the findings were mixed as some reported that women had worse well-being (e.g., [89,90]), whereas others showed that gender was not related to well-being (e.g., [67,91]).

Overall, older workers had better well-being than younger workers, as demonstrated by 33 of the 47 articles statistically investigating the relationship between age and well-being. For example, younger physicians in Turkey had higher scores on depression, anxiety, and stress [92]. This finding seems to replicate across similar crises and countries worldwide. During the SARS epidemic in China, for example, younger workers experienced more depressive symptoms [91]. Younger workers were found to have worse well-being during H1N1 in Japan [93], SARS in Canada [89] and Singapore [94], as well as COVID-19 in Spain [95], for example. However, just over a third of the articles (35.53%) that explored the relationship between age and well-being did not find support for a relationship.

Research was mixed on whether education is related to well-being. Twenty-six of the 41 articles reported that education was unrelated to worker well-being, whereas 15 reported that a relationship was present. Most reported that workers with more education had better well-being than those with less education. This relationship was replicated by researchers exploring the well-being of healthcare workers in China and other Asian countries during SARS and COVID-19, for example (e.g., [67,70,96]). However, four articles found that less education was related to better well-being.

#### 4.5.2 Social-related factors

Social-related factors include the relationships present in workers’ personal lives (e.g., parent-child, siblings) or at work (e.g., worker-manager, caregiver-patient). Seventy-seven (41.18%) retained articles explored at least one social-related factor, with marriage (*n* = 51), parenthood (*n* = 19), and social support (*n* = 9) being the most frequently explored factors.

Research was mixed on whether being married is related to better worker well-being. Most authors explored well-being differences between married and non-married participants. Some used other terms, such as partner(ed). These terms are combined in the “married” category. Of the 21 articles that showed a significant relationship between marriage and well-being, seven found that married workers had better well-being than those not married, whereas six articles found that those who were not married had better well-being than their married counterparts (the remaining articles were conflicting or miscellaneous). Most of the retained articles, however, did not find a relationship between marital status and well-being (*n* = 30). Interestingly, marital status was significant in some studies and not others, even within a single pandemic/epidemic, general occupational group, and region in which the study is contextualized. For example, married healthcare workers in China during COVID-19 had worse anxiety and depressive symptoms than those not married [70]. However, Xiao et al. [87] did not find a relationship between marital status and stress among healthcare workers in China during COVID-19.

Research was mixed on whether parents had better worker well-being. Of the 19 articles exploring the relationship between parenthood and workers' well-being, 12 reported that no relationship was present. Of the articles that did not find support for a significant relationship between parenthood and well-being, most were within the context of COVID-19 (e.g., [6,[11], [12], [13]]) and a few were in the context of MERS and SARS (e.g., [97,98], respectively). The number of articles conducted in various countries during COVID-19 suggests that the pandemic is not related to parents’ well-being differently than workers with no children. Similarly, most studies conducted on past crises did not find a relationship between parenthood and well-being.

Workers with (more) social support seem to have better well-being than workers with no, or inadequate, support. Eight articles that reported a relationship between social support and well-being found this; the others did not use significance testing to explore the relationship or report conflicting findings. Overall, the studies were all conducted with Asian workers during COVID-19 (e.g., [8,86]). For example, increased social support was related to decreased COVID-19-related anxiety [8]. Although most articles explored healthcare workers’ experiences, social support also appeared beneficial for other workers in a general sample, which included workers in non-healthcare-related occupational groups (e.g., community workers, police, journalists), during COVID-19 [86]. However, these occupational groups were combined with healthcare workers for the statistical analyses. Although not as commonly explored, some articles also suggest that social support also helped Asian workers during SARS (e.g., [63,90]).

#### 4.5.3 Workplace-related factors

Workplace-related factors are constructs related to participants' work(place), including factors attributable to the workplace (e.g., work setting, communications), and the role(s) that workers play within companies and institutions (e.g., occupation, seniority). One hundred and forty-seven (78.61%) of the retained articles explored at least one workplace-related factor. Workers’ occupation (*n* = 71), seniority or experience (*n* = 16), and frontline worker status (*n* = 14) were explored the most.

Overall, workers' occupation was related to their well-being (Table 4). Of the 71 articles exploring this relationship, 74.65% (*n* = 53) reported that a relationship was present. The remaining 18 articles did not find a relationship. The relationship between occupation and workers’ well-being in the retained articles was primarily explored by comparing multiple *general* (i.e., comparing large occupational work domains) or *specific* occupational groups (i.e., comparing specific jobs within occupational domains). Articles explored specific occupational groups more often than general occupational groups. Regarding the general occupational groups, healthcare workers typically had worse well-being than non-healthcare workers. Non-healthcare workers typically had better well-being than other groups. With regards to comparisons between specific health-care occupations, doctors typically had better well-being than other specific occupational groups. Nurses, in contrast, had worse well-being than other occupational groups. Students who only comprised academic positions with an occupational component (e.g., medical or PhD students) typically had worse well-being than other occupational groups. Findings regarding (non-)medical, technical, administrative, and emergency occupational groups were only explored one or two times within the retained articles.

**Table 4:** The number of articles investigating workers’ occupation and their well-being.

	Not related	Related	Worse well-being	Better well-being	No significance test	Mixed	Conflicting
**General occupational groups**							
Healthcare workers	2	12	5	1	0	1	5
Non-healthcare workers	2	9	1	4	0	1	3
Medical workers	0	2	0	0	0	0	2
Non-medical workers	0	2	0	0	0	0	2
Technical staff	0	1	0	0	0	0	1
Administrative staff	0	1	0	0	0	0	1
Emergency staff	0	1	0	1	0	0	0
**Specific occupational groups**							
Doctor	10	36	4	11	3	2	16
Nurse	11	39	10	3	3	2	21
Tech	2	6	1	0	0	0	5
Administration	2	11	1	2	2	0	6
Pharmacist	0	4	1	1	0	0	2
Physiotherapist	1	3	0	1	0	0	2
Allied Health Professional	0	4	0	0	0	0	4
Student (e.g., medical, PhD)	0	7	4	0	0	0	3
Other	14	40	4	10	3	0	23

*Note*. The number of articles exploring different occupations exceeds the total number mentioned in Table 3 as the articles reported findings regarding more than one occupation. Conflicting results are when multiple statistical tests are used to explore the relationship between the factor and one or more well-being constructs and the pattern of the results is not the same. Miscellaneous findings are patterns of results that are curvilinear or when there is an interaction tested that cannot be disentangled. No significance test refers to findings that did not use a statistical test to explore the relationship between the factor and well-being or did not report statistical tests allowing to identify which specific groups were different from one another.

Research was mixed on whether seniority or relevant experience during pandemics and epidemics was related to workers' well-being. Of the 16 articles exploring this relationship, 37.50% did not find support for it, and they were situated in many countries and pandemics and epidemics. Four articles were situated during COVID-19 and explored workers’ well-being in Palestine [99], Turkey [100], China [101], and Singapore [102]. Two articles were also situated in Singapore during SARS [103] and South Korea during MERS [97]. Ten articles, however, found a relationship between seniority or relevant experience and workers’ well-being. Four of the ten reported that more experience was related to better well-being than those with less [[64], [87], [104], [105]]; three reported that less experience was related to better well-being than those with more [[84], [106], [107]].

Frontline (vs. non-frontline) workers experienced worse well-being. Of the 14 retained articles that explored a relationship between working on the frontline and well-being, 85.71% (*n* = 12) showed this pattern. Seven of the 12 articles (58.33%) described that frontline (vs. non-frontline) healthcare workers in China experienced worse well-being [13,96,[108], [109], [110], [111], [112]]. For example, frontline healthcare workers in China experienced more severe depression-, anxiety-, and distress-related symptoms [109]. Evidence suggests that frontline workers in other countries, primarily the Middle East, also experienced worse well-being during COVID-19. This includes workers in Turkey [92], Saudi Arabia [113], Iran [114], and Italy [115].

#### 4.5.4 Pandemic-related factors

The most distal of the categories, pandemic-related factors encompass factors that are either a) relevant in the context of a pandemic or epidemic (e.g., personal protective equipment [PPE] access, risk of exposure), and/or b) a direct result of one of these public health crises (e.g., quarantining, symptoms). Ninety-eight (52.41%) of the retained articles explored at least one pandemic-related factor. Risk or exposure (*n* = 22), known infection or death in workers’ close circle (*n* = 16), and PPE access (*n* = 14) were the most explored factors.

Higher risk of becoming ill or exposure to the pathogen was related to worse well-being. Of the 22 retained articles that reported a relationship between risk or exposure and well-being, 68.18 % (*n* = 15) show this finding. For example, high risk of contracting SARS was related to chronic stress, higher depression levels, and anxiety in healthcare workers in Hong Kong [9]. Here, infection risk was based on the type of medicine being practiced by healthcare workers. Those practicing respiratory medicine were categorized as high-risk, whereas those practicing non-respiratory medicine were low-risk. These findings were similar for other healthcare workers in Asia during/after SARS (e.g., [91,103]) and COVID-19 [12,60,116,117]. Although not explored in other regions to the same degree, some studies suggest that this relationship may be the same for healthcare workers during SARS or COVID-19 in Europe [95,115] and Canada [89,98].

Workers who did not know anyone in their close circle (e.g., family, co-workers) who caught the pathogen or died from it had better well-being than those who did. Of the 13 retained articles that reported a relationship between known infection or death in workers' close circle and well-being, 69.23% (*n* = 9) showed this relationship. For example, healthcare workers in Italy who knew a colleague who had been infected and was in quarantine during COVID-19 had worse stress, post-traumatic stress, and depression [115]. The workers who knew a co-worker who died from the virus also experienced worse post-traumatic stress and depression. Similarly, healthcare workers in China were more vulnerable to anxiety if their co-workers had COVID-19 [118]. Multiple researchers reported similar findings regarding a friend or family member affected (e.g., [[119], [120]]). Healthcare workers in the US with immediate family members sick with COVID-19 expressed higher personal/family and career stress compared to those without infected family members [120]. Cheng et al. [119] found that having family members whom SARS killed was related to worsened workers’ depressive symptoms.

Having adequate (or more) PPE access was seemingly related to better well-being. Of the 12 retained articles that reported a relationship between PPE access and well-being, 41.67% (*n* = 5) reported this relationship. However, 50.00% (*n* = 6) reported miscellaneous findings or did not use significance testing. For example, PPE access was related to lower distress and higher job satisfaction in healthcare workers working in Iranian public and private hospitals during COVID-19's peak [[11], [12], [13]]. This positive relationship between PPE access and well-being was found during COVID-19 in China [121,122], Poland [123,124], and Israel [10]. Additionally, appropriate protection against infection was related to lower psychopathological symptoms for diverse medical workers (e.g., physiotherapists, midwives; [123]).

### 4.6 Precarious employment

Only 19 articles explored one or more dimension of precarious employment, with 11 exploring job insecurity, 14 exploring income inadequacy, and two exploring limited rights and protection. The studies showed that several workers experienced precarious employment in the context of, or due to, such crises. Of the 19 articles, 12, all in the context of COVID-19, explored how one or more dimensions of precarious employment related to workers’ well-being using statistical testing. Eight used statistical testing to investigate the relationships between well-being and income inadequacy, eight investigated its relationship to job insecurity, and none explored how it related to lack of rights and protection.

Table 5 shares the findings from the 10 articles that showed that precarious employment was related to poorer worker well-being during COVID-19. This relationship also appeared cross-culturally. For example, studies reported how dimensions relating to job insecurity were associated with higher negative well-being [[76], [125], [126], [127]]. This pattern of results was found in workers residing in Kuwait [125], the US [76], and Israel [126]. The association between income inadequacy and worker well-being provides a similar conclusion. Workers reporting less income satisfaction or more financial hardship reported poorer well-being. Nevertheless, two articles exploring a construct related to income inadequacy showed no (or a mixed) relationship. Three articles focused on the interplay between precariousness, well-being and third variables (e.g., alcohol use) [[55], [128], [129]]. These results suggest that experiencing these forms of precarious employment may not always be directly related to well-being but rather may be related to an aspect of workers’ lives, which, in turn, is related to poorer well-being.

**Table 5:** Summary of retained articles that explored the association between precarious employment and well-being.

**Citation**	**Country**	***N***	**Design**	**Findings**

*No/mixed association with well-being*				
[130]	Singapore	258	CS	73.00% were employed full-time; 6.00% were casually/temporarily employed. Families had an income higher than half of the country's households. Results regarding the relationships between employment/income and well-being were mixed.
[131]	Turkey	200	CS	69.00% were satisfied with their income. Income satisfaction was not related to well-being.
*Direct negative association with well-being*				
[125]	Kuwait	1,018	CS	4.90% of participants were worried about losing their job.
[132]	Thailand	300	CS	Job uncertainty was positively related to worker burnout.
[127]	US	10,368	CS	Laid-off/furloughed (vs. working) participants had higher COVID-19 fear.
[76]	US	645	L	Most participants reported job loss and income reduction for themselves and their families after COVID-19 began, which were related to worse well-being.
[133]	US	1,996	CS	Most had enough money, with a little left over. Finances and distress were negatively related.
[126]	Israel	293	CS	56.00% of furloughed participants reported nervousness and anxiety, compared to 26.00% of those unemployed. Furloughed participants had higher distress than unemployed or full-time workers, but not part-time workers.
[134]	US	474	CS	Approximately one-third reported concern regarding their finances in the next year, and 80.50% experienced worry regarding their employment due to COVID-19.
*Other*				
[128]	Brazil	536	CS	Physicians, nurses, and dentists experienced COVID-19-related job loss. Dentists experienced a lower number of jobs. Physicians had the highest monthly income. All three groups reported significant income reductions. Physicians reported the lowest levels of anxiety and thoughts about giving up the profession, whereas dentists reported the highest.
[129]	Pakistan	50	CS	42.00% of lab professionals agreed that there is a threat to their employment during the pandemic and constantly fear being laid off if financial hardships were to continue.
[55]	Canada	320	CS	56.60% reported work hours changing during COVID-19. 55.00% reported no change in income since COVID-19 started; 19.40% reported a 100.00% loss. Income loss was associated with alcohol problems through increased alcohol use.

*Note.* All studies were in the context of COVID-19. ID = identification number; *N* = sample size; CS = cross-sectional; L = longitudinal.

## 5 Discussion

We found that, during epidemics and pandemics, negative well-being was typically not experienced frequently, and, in terms of levels, was rarely experienced more than at moderate levels on average. Positive well-being was typically experienced at moderate to high levels overall and positive well-being manifestations were usually experienced quite frequently. We also found several factors related to workers' well-being during such crises. The most frequently examined factors related to workers themselves, their social relationships, their work, and pandemics and epidemics were explored in-depth. Age, gender, social support, occupation, frontline status, risk/exposure, knowing someone infected or killed by the virus, and PPE access showed a clear relationship with well-being. Most articles exploring precarious employment, although limited in number, indicated a deleterious relationship with workers’ well-being.

### 5.1 Well-being during pandemics and epidemics

To our knowledge, this is the first review to adopt a holistic perspective of workers' well-being. None of the previously published reviews have provided a clear picture of the published research on well-being considering workers’ positive general well-being or work-related well-being. Across the 187 retained articles, we found that general positive well-being and work-related well-being were experienced at high levels or very frequently. In the case of positive well-being, it was often experienced more than negative well-being. In addition to these findings, negative well-being was found to be adversely related to the presence of certain pandemics and epidemics. For example, workers typically experienced low to moderate negative well-being during SARS and COVID-19 outbreaks. However, workers typically had high negative well-being during less frequently explored pandemics and epidemics (e.g., H1N1, MERS). It is of importance to note that only ten articles on negative well-being were contextualized during these less frequently explored crises. Across these crises, indicators of general positive and work-related well-being were experienced moderately to highly *or* were experienced quite frequently. These findings suggest that workers were still able to experience prospering in their lives and at work during such crises.

Our findings show a positive outlook, as most workers seemed to have adapted well to virus-related events. Such positive adaptation highlights humans' resilience in the darkest of times. As highlighted by Bonanno [135], many individuals function normally and experience positive emotional experiences after experiencing loss or traumatic events. This positive adaptation is reminiscent of other survivors' experiences after tragic events. For example, even though rates of post-traumatic stress disorder were higher in survivors of the Alexander L. Kielland Disaster (13.70%) in the following year after the crisis than in a matched community sample (1.10%), most survivors were not experiencing any maladaptive psychological or physical functioning during this period ([135]; also see [136]). Nevertheless, and like a proportion of the survivors of the oil rig disaster, some workers do suffer during pandemics and epidemics. More recent research that is not included in this scoping review due to being published after our cut-off date supports this notion. Research from the UK and France showed that although most workers were prospering to some degree (67% moderately positive, 8% flourishing) during the COVID-19 lockdown, 18% were languishing (higher negative well-being than positive [137]). Relevant to this smaller proportion of languishing workers, as discussed by Wong et al. [138], such suffering may be caused by several pandemic/epidemic-related consequences (e.g., lack of meaning or purpose, loss of social connectedness, thoughts of death). Referencing positive psychology's second wave (PP2.0), Wong et al. [138] suggests that one can convert such existential languishing into prospering; however, different ways of coping and interventions should be developed to aid in such transition. Based on our findings, developing person-specific interventions based on factors that contribute to lower well-being could aid in transforming existential languishing in prospering.

### 5.2 What contributes to higher or lower well-being?

**Fig. 2:**
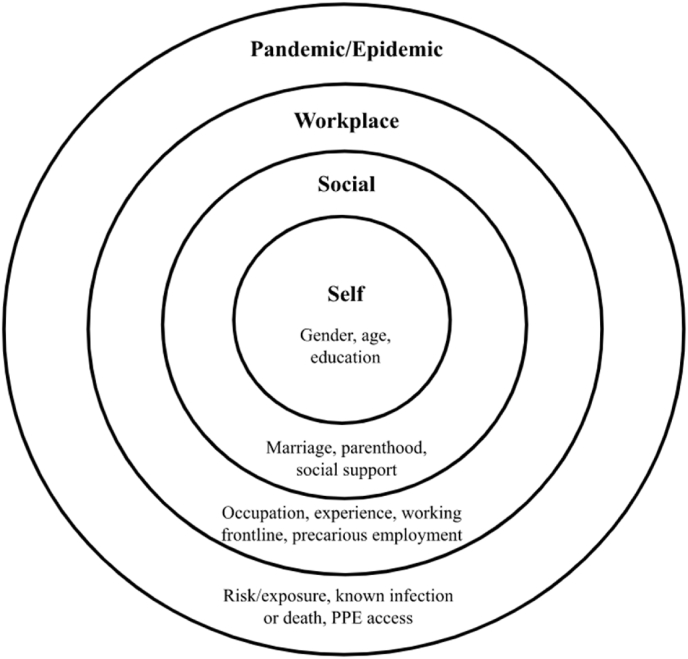
An ecological perspective of the explored risk and resilience factors.

Inspired by the third wave of positive psychology, multiple theories within psychology and public health were married to holistically consider factors that were related to workers’ well-being during pandemics and epidemics. We integrated the following theories: precarious work within industrial-organizational psychology [34], social determinants of mental health within public health [20], and ecological systems theory within developmental and community psychology [21]. As seen in Fig. 2, risk and resilience factors exist at different ecological levels that range in proximity to workers. Proximal factors are those related to the self and interpersonal relationships; distal factors are related to the workplace and the pandemic or epidemic. Although some showed a clear relationship with well-being, others did not. To our knowledge, this is the first time ecological systems theory has been used in a review to better understand worker well-being during pandemics and epidemics. Only a few empirical studies have used Ecological Systems Theory to better understand factors that impact Canadian workers during COVID-19 [[139], [140], [141]].

The three most explored self-related factors were gender, age, and education. Men and older workers typically had better well-being; education was not consistently related to well-being. These findings are aligned with recent research showing that women (e.g., [[142], [143], [144]]) and younger individuals (e.g., [145,146]) experienced adverse well-being during COVID-19. These studies, and our findings, suggest that women and younger workers may need more well-being-related support. Interestingly, women and younger workers are also more likely to be in precarious employment in Canada [147]. It may be beneficial to provide services within these workers’ general lives and workplace(s). In their general lives, policies making mental health services more accessible could be an avenue. Alternatively, stakeholders should promote programs specific to women or youths. Regarding their workplace, employers can implement policies that provide more mental health benefits.

The three most explored social-related factors were marital status, parenthood, and social support. Marital status’ relationship with well-being was mixed or not present. Those reporting a relationship showed that married individuals had better well-being during virus-related crises. Parenthood appeared to be unrelated to workers' well-being, as 63.16% of the articles reported no relationship being present. For social support, however, there was some evidence that receiving social support was related to more prospering. Establishing a relationship between social-related factors and well-being may be harder as additional variables may explain or confound the relationship. For example, parenthood may not be related to well-being, but access to childcare and sufficient income to support children may. Newer research on workers’ experiences during COVID-19 has shown that concerns regarding childcare were related to poorer well-being (e.g., [148]). Additionally, these factors may interact with one another. For example, being a parent may be associated with more prospering well-being if the worker receives more social support (as shown in non-pandemic circumstances; [149]). Interestingly, familial support was the most frequently explored form of social support, but there was a paucity of research on possible social support at work (e.g., support from colleagues or manager). Future research should explore whether social support from workplace colleagues and superiors can protect workers from the negative aspects of such crises.

The three most explored workplace-related factors were occupation, seniority or experience, and frontline status. Occupation and frontline status were related to well-being; research was mixed on whether seniority or experience was related to workers’ well-being. Healthcare workers (generally), nurses, and (healthcare) students typically had worse well-being than other (general) occupational groups. Doctors and non-healthcare workers (generally) had better well-being than those in other occupations. Frontline (vs. non-frontline) workers typically had worse well-being. Interestingly, the factors associated with working near the pathogen emerged as related to well-being. For example, healthcare workers, regardless of whether they were explored as general or specific occupational groups, experienced worse well-being than non-healthcare workers. Mushtaq and colleagues [150] showed that well-being issues arose from increased job stress and fear of spreading the disease to close others, for example. With respect to nurses, Matsuishi et al. [93] showed that they had higher post-traumatic stress scores than doctors shortly after H1N1 affected Kobe City, Japan, in 2009. This phenomenon occurred across crises as a more recent rapid review conducted during COVID-19 showed some evidence that nurses may have worse well-being than doctors [151]. Nurses are still often considered marginal and unimportant members of the healthcare team, and their professional autonomy is not accepted by some doctors [152]. Although we aimed to explore diverse occupational groups, researchers primarily studied healthcare workers. Other occupational groups also experienced poorer well-being, however, warranting more exploration.

The three most explored pandemic-related factors were risk or exposure, known infection or death in one's close circle, and PPE access. All three factors were related to well-being. Those with higher risk or exposure, known infection or death within their close circle, or limited PPE access typically experienced poorer well-being than their counterparts. It is argued that inequalities marginalized workers experienced before the crisis may be intensified by these pandemic-related risk factors, possibly leading to poorer physical health *and* well-being among these workers. These findings are supported by more recent research that has been published on workers' well-being during COVID-19 (e.g., [[153], [154], [155], [156]]). Although we showed that PPE access is associated with better well-being, special attention is needed regarding *wearing* such equipment. For example, Radha et al. [157] show that healthcare workers (i.e., a front-line occupational group that wore PPE for prolonged periods during COVID-19) experienced several physical (e.g., skin lesions, headaches, fatigue) and psychological (e.g., anxiety, fear) problems with regards to wearing PPE. Policies focused on equity should be implemented in workplaces and communities to curb infection rates and support *all* workers' well-being. We show that specific workers (e.g., women, younger workers) and occupational groups (healthcare workers, nurses, students) reported worse well-being and would benefit the most from well-being-related policies.

### 5.3 The relationship between precarious employment and worker well-being

Although research exploring the relationship between precarious employment and well-being is limited, it is apparent that income inadequacy and job insecurity are related to poorer worker well-being. In addition, the fact that precarious employment was found to be related to poorer well-being in different countries (e.g., Pakistan, Canada, Brazil, Israel) suggests that it is not culturally dependent. The relationship found in our review between precarious employment and worker well-being echoes other reviews [158] that show how those in precarious employment situations (e.g., young people, women, low-skilled and temporary workers) experienced the most economic consequences during COVID-19. As instantaneous economic hardship during a COVID-19 lockdown was associated with worse negative well-being (depression, health anxiety) [159], it is apparent how pandemics and epidemics may disproportionately affect precarious workers. The fact that younger workers and women are more likely to be found in precarious employment situations may partly explain our findings in that identifying as a woman and being younger in age was related with worse well-being.

A scarcity of research explores how a lack of rights and protection (the third dimension of precarious employment) relates to worker well-being. A lack of rights and protection refers to lower unionization rates, social security, and workplace rights [34]. This unexplored area of precarious employment is important to examine during pandemics and epidemics as some pre-crisis research shows how workers' rights and protection are positively associated with better well-being in general and at work (e.g., [160]). Understanding how workers’ rights and protections is related to their well-being has many implications. For example, if research during these crises shows similar findings in that better rights and protections is related to better well-being, it would provide evidence to policymakers and employers that addressing this dimension of precarious employment is worthwhile.

### 5.4 Limitations

Only research published at the beginning of COVID-19 was considered, limiting our ability to speak to workers' well-being later in the pandemic and after its conclusion. As such, our COVID-19-related findings should only be considered representative of workers' early experiences during COVID-19. Future reviews are encouraged to use our findings as a steppingstone to better understanding workers' experiences later during the COVID-19 pandemic and its resolution. Similarly, the scoping review did not explore other public health events (e.g., HIV/AIDS, natural disasters). Of these other public health events, scholars working on future reviews are implored to give special attention to the HIV/AIDS epidemic and how its relationship with workers' well-being compares to that of other virus-related epidemics and epidemics. Third, these results cannot be generalized to all workers worldwide during every pandemic and epidemic. Little is known on crises before COVID-19, non-healthcare workers in some continents (e.g., Africa, South America), and workers experiencing precarious employment. Fourth, suffering workers may not take part in studies like the ones included in this review, which may bias the findings. Fifth, business-oriented databases were not used to retrieve potentially relevant articles, possibly leaving unexplored some work-related outcomes (e.g., work attitudes, objective measures of performance) relevant to workers' experiences and well-being during pandemics and epidemics. Sixth, none of the retained articles investigated how biological factors (e.g., cortisol levels, heart rate variability) were related to worker well-being during these crises. As Bronfenbrenner and Morris [161] later conceptualized genetic and physical biological dispositions as part of the ontosystem (i.e., the “self” level in this article; also see: [162]), it would be of interest to further explore how genetic dispositions and processes may be related to workers' well-being during pandemics and epidemics. Lastly, a single method of coding descriptive findings was used to identify emerging trends related to workers' well-being in different contexts. With this, some nuances underlying the studies’ findings may have been lost.

## 6 Conclusion

Despite the aforementioned limitations, and unlike other reviews, this scoping review simultaneously explored workers’ general and work-specific well-being during pandemics and epidemics. For academics, these findings can be a foundation for future studies examining worker well-being during future crises. There are also implications for policy advisors, mental health and community organizations, and workplace stakeholders (e.g., union organizers, human resources). With a sense of how pandemic and epidemic contexts are related disproportionally with the well-being of some workers compared to others, stakeholders should focus on equity-based solutions to help those suffering the most during future crises.

## Funding declaration

TP has received funding from 10.13039/501100004489Mitacs (IT19992) and the 10.13039/100021638Social Sciences and Humanities Research Council (752-2021-2470) to complete this research. The authors certify that these organizations were not involved in any stage of the research and publication process.

## CRediT authorship contribution statement

**Tyler Pacheco:** Writing – review & editing, Writing – original draft, Validation, Supervision, Project administration, Methodology, Investigation, Funding acquisition, Formal analysis, Data curation, Conceptualization. **Simon Coulombe:** Writing – review & editing, Validation, Supervision, Resources, Project administration, Methodology, Investigation, Funding acquisition, Formal analysis, Data curation, Conceptualization. **Nancy L. Kocovski:** Writing – review & editing, Validation, Supervision, Project administration, Methodology, Investigation, Formal analysis, Data curation, Conceptualization. **Julia Carbone:** Writing – review & editing, Validation, Methodology, Investigation, Formal analysis, Data curation, Conceptualization.

## Declaration of competing interest

On behalf of all authors, the corresponding author states that there is no conflict of interests.

## Footnotes

3 Although we explore worker well-being during *viral* pandemics and epidemics, we henceforth refer to these crises as “pandemics” and “epidemics” for brevity.
