# Supplementary material for: Workers’ well-being during viral pandemics and epidemics: A scoping review
Source: Compr Psychoneuroendocrinol. 2025 Mar 4;22:100286. doi: 10.1016/j.cpnec.2025.100286 (PMC12126619; doi:10.1016/j.cpnec.2025.100286)
Supplement: Multimedia component 1 [file mmc1.docx]

**References for Retained Articles**

Ahmed, F., Zhao, F., & Faraz, N. A. (2020). How and when does inclusive leadership curb

psychological distress during a crisis? Evidence from the COVID-19 outbreak. *Frontiers in Psychology*, *11*, 1-13. https://doi.org/10.3389/fpsyg.2020.01898

Aksoy, Y. E., & Koçak, V. (2020). Psychological effects of nurses and midwives due to

COVID-19 outbreak: The case of Turkey. *Archives of Psychiatric Nursing*, *34*(5), 427-433. https://doi.org/10.1016/j.apnu.2020.07.011

Al-Hanawi, M. K., Mwale, M. L., Alshareef, N., Qattan, A. M. N., Angawi, K., Almubark, R., &

Alsharqi, O. (2020). Psychological distress amongst health workers and the general public during the COVID-19 pandemic in Saudi Arabia. *Risk Management and Healthcare Policy*, *13,* 733-742. http://doi.org/10.2147/RMHP.S264037

Al-Hashel, J. Y., & Ismail, I. I. (2020). Impact of coronavirus disease 2019 (COVID-19)

pandemic on patients with migraine: A web-based survey study. *The Journal of Headache and Pain*, *21*, 1-9. https://doi.org/10.1186/s10194-020-01183-6

Alkhamees, A. A., Alrashed, S. A., Alzunaydi, A. A., Almohimeed, A. S., & Aljohani, M. S.

(2020). The psychological impact of COVID-19 pandemic on the general population of Saudi Arabia. *Comprehensive Psychiatry*, *102*, 1-9. https://doi.org/10.1016/j.comppsych.2020.152192

Almaghrabi, R. H., Alfaradi, H., Al Hebshi, W. A., & Albaadani, M. M. (2020). Healthcare

workers experience in dealing with Coronavirus (COVID-19) pandemic. *Saudi Medical Journal*, *41*(6), 657-660. https://doi.org/10.15537/smj.2020.6.25101

Alonazi, W. B. (2020). The impact of emotional intelligence on job performance during

COVID-19 crisis: A cross-sectional analysis. *Psychology Research and Behavior Management*, *13*, 749-757. https://doi.org/10.2147/PRBM.S263656

Alruwaili, N. (2015). *Respiratory therapists’ knowledge, skills, and attitudes regarding*

*MERS-CoV disasters* [Master’s thesis]. Georgia State University. https://scholarworks.gsu.edu/rt_theses/26

Al Sulais, E., Mosli, M., & AlAmeel, T. (2020). The psychological impact of COVID-19

pandemic on physicians in Saudi Arabia: A cross-sectional study. *Saudi Journal of Gastroenterology: Official Journal of the Saudi Gastroenterology Association*, *26*(5), 249-255. https://doi.org/10.4103/sjg.SJG_174_20

Anicich, E. M., Foulk, T. A., Osborne, M. R., Gale, J., & Schaerer, M. (2020). Getting back to

the “new normal”: Autonomy restoration during a global pandemic. *Journal of Applied Psychology*, *105*(9), 931-943. http://dx.doi.org/10.1037/apl0000655

Apisarnthanarak, A., Apisarnthanarak, P., Siripraparat, C., Saengaram, P., Leeprechanon, N., &

Weber, D. J. (2020). Impact of anxiety and fear for COVID-19 toward infection control practices among Thai healthcare workers. *Infection Control & Hospital Epidemiology*, *41*(9), 1093-1094. https://doi.org/10.1017/ice.2020.280

Ayub, M., Arshad, D., Maqbool, N., Zahid, M., Malik, R. S., Rizvi, Z. A., Arshad, U., & Khan,

S. U. (2020). Physicians' attitudes towards treating patients in the context of COVID-19 pandemic in Pakistan. *Cureus*, *12*(9), 1-8. https://doi.org/10.7759/cureus.10331

Bai, Y., Lin, C. -C., Lin, C. -Y., Chen, J. -Y., Chue, C. -M., & Chou, P. (2004). Survey of stress

reactions among health care workers involved with the SARS outbreak. *Psychiatric Services*, *55*(9), 1055-1057. https://doi.org/10.1176/appi.ps.55.9.1055

Barbato, M., & Thomas, J. (2020). Far from the eyes, close to the heart: Psychological Impact of

COVID-19 in a Sample of Italian foreign workers. *Psychiatry Research*, *290*, 1-2. https://doi.org/10.1016/j.psychres.2020.113113

Bender, W. R., Srinivas, S., Coutifaris, P., Acker, A., & Hirshberg, A. (2020). The psychological

experience of obstetric patients and health care workers after implementation of universal SARS-CoV-2 testing. *American Journal of Perinatology*, *37*(12), 1271-1279. https://doi.org/10.1055/s-0040-1715505

Bettinsoli, M. L., Di Riso, D., Napier, J. L., Moretti, L., Bettinsoli, P., Delmedico, M., Piazolla,

A., & Moretti, B. (2020). Mental health conditions of Italian healthcare professionals during the COVID‐19 disease outbreak. *Applied Psychology: Health and Well‐Being*, *12*(4), 1054-1073. https://doi.org/10.1111/aphw.12239

Bostan, S., Akbolat, M., Kaya, A., Ozata, M., & Gunes, D. (2020). Assessments of anxiety levels

and working conditions of health employees working in COVİD-19 pandemic hospitals. *Electronic Journal of General Medicine, 17*(5), 1-5. https://doi.org/10.29333/ejgm/8228

Butsashvili, M., Triner, W., Kamkamidze, G., Kajaia, M., & McNutt, L. -A. (2007). Knowledge

and anticipated behavior of health care workers in response to an outbreak of pandemic influenza in Georgia. *The Journal of Infection in Developing Countries*, *1*(3), 329-332. https://doi.org/10.3855/jidc.373

Cai, Q., Feng, H., Huang, J., Wang, M., Wang, Q., Lu, X., Xie, Y., Wang, X., Liu, Z., Hou, B.,

Ouyang, K., Pan, J., Li, Q., Fu, B., Deng, Y., & Liu, Y. (2020). The mental health of frontline and non-frontline medical workers during the coronavirus disease 2019 (COVID-19) outbreak in China: A case-control study. *Journal of Affective Disorders*, *275*, 210-215. https://doi.org/10.1016/j.jad.2020.06.031

Carroll, N., Sadowski, A., Laila, A., Hruska, V., Nixon, M., Ma, D. W. L., Haines, J., & Guelph

Family Health Study. (2020). The impact of COVID-19 on health behavior, stress, financial and food security among middle to high income Canadian families with young children. *Nutrients*, *12*(8), 1-14. https://doi.org/10.3390/nu12082352

Cerami, C., Santi, G. C., Galandra, C., Dodich, A., Cappa, S. F., Vecchi, T., & Crespi, C. (2020).

Covid-19 outbreak in Italy: Are we ready for the psychosocial and the economic crisis? Baseline findings from the PsyCovid study. *Frontiers in Psychiatry*, *11*, 1-9. https://doi.org/10.3389/fpsyt.2020.00556

Chan, A. O. M., & Huak, C. Y. (2004). Psychological impact of the 2003 severe acute

respiratory syndrome outbreak on health care workers in a medium size regional general hospital in Singapore. *Occupational Medicine*, *54*(3), 190-196. https://doi.org/10.1093/occmed/kqh027

Chan, S. S. C., Leung, G. M., Tiwari, A. F. Y., Salili, F., Leung, S. S. K., Wong, D. C. N.,

Wong, A. S. F., Lai, A. S. F., & Lam, T. H. (2005). The impact of work-related risk on nurses during the SARS outbreak in Hong Kong. *Family and Community Health*, *28*(3), 274-287.

Charoensukmongkol, P., & Phungsoonthorn, T. (2021). The effectiveness of supervisor support

in lessening perceived uncertainties and emotional exhaustion of university employees during the COVID-19 crisis: The constraining role of organizational intransigence. *The Journal of General Psychology*, *148*(4), 431-450. https://doi.org/10.1080/00221309.2020.1795613 *

Chen, H., Sun, L., Du, Z., Zhao, L., & Wang, L. (2020). A cross‐sectional study of mental health

status and self‐psychological adjustment in nurses who supported Wuhan for fighting against the COVID‐19. *Journal of Clinical Nursing*, *29*(21-22), 4161-4170. https://doi.org/10.1111/jocn.15444

Chen, J., Liu, X., Wang, D., Jin, Y., He, M., Ma, Y., Zhao, X., Song, S., Zhang, L., Xiang, X.,

Yang, L., Song, J., Bai, T., & Hou, X. (2021). Risk factors for depression and anxiety in healthcare workers deployed during the COVID-19 outbreak in China. *Social Psychiatry and Psychiatric Epidemiology*, *56*, 47-55. https://doi.org/10.1007/s00127-020-01954-1 *

Chen, N. -H., Wang, P. -C., Hsieh, M. -J., Huang, C. -C., Kao, K. -C., Chen, Y. -H., & Tsai, Y.

-H. (2007). Impact of severe acute respiratory syndrome care on the general health status of healthcare workers in Taiwan. *Infection Control & Hospital Epidemiology*, *28*(1), 75-79. https://doi.org/10.1086/508824

Cheng, S. K. W., Sheng, B., Lau, K. K., Wong, C. W., Ng, Y. K., Li, H. L., Chan, E. L. Y., Tso,

E. Y. K., Lam, K., Chau, T. N., & Chiu, M. C. (2004). Adjustment outcomes in Chinese patients following one-month recovery from severe acute respiratory syndrome in Hong Kong. *The Journal of Nervous and Mental Disease*, *192*(12), 868-871. https://doi.org/10.1097/01.nmd.0000147169.03998.dc

Cheng, S. K. W., Wong, C. W., Tsang, J., & Wong, K. C. (2004). Psychological distress and

negative appraisals in survivors of severe acute respiratory syndrome (SARS). *Psychological Medicine*, *34*(7), 1187-1195. https://doi.org/10.1017/S0033291704002272

Chew, N. W. S., Lee, G. K. H., Tan, B. Y. Q., Jing, M., Goh, Y., Ngiam, N. J. H., Yeo, L. L. L.,

Ahmad, A., Khan, F. A., Shanmugam, G. N., Sharma, A. K., Komalkumar, R. N., Meenakshi, P. V., Shah, K., Patel, B., Chan, B. P. L., Sunny, S., Chandra, B., Ong, J. J. Y., ... & Sharma, V. K. (2020). A multinational, multicentre study on the psychological outcomes and associated physical symptoms amongst healthcare workers during COVID-19 outbreak. *Brain, Behavior, and Immunity*, *88*, 559-565. https://doi.org/10.1016/j.bbi.2020.04.049

Chew, N. W. S., Ngiam, J. N., Tan, B. Y. -Q., Tham, S. -M., Tan, C. Y. -S., Jing, M.,

Sagayanathan, R., Chen, J. T., Wong, L. Y. H., Ahmad, A., Khan, F. A., Marmin, M., Hassan, F. B., Sharon, T. M. -L., Lim, C. H., Mohaini, M. I. B., Danuaji, R., Nguyen, T. H., Tsivgoulis, G., … Sharma, V. (2020). Asian-Pacific perspective on the psychological well-being of healthcare workers during the evolution of the COVID-19 pandemic. *BJPsych Open,* *6*(6), 1-11. https://doi.org/10.1192/bjo.2020.98

Chong, M. -Y., Wang, W. -C., Hsieh, W. -C., Lee, C. -Y., Chiu, N. -M., Yeh, W. -C., Huang, T.

-L., Wen, J. -K., Chen, C. -L. (2004). Psychological impact of severe acute respiratory syndrome on health workers in a tertiary hospital. *The British Journal of Psychiatry,* *185*(2), 127-133. https://doi.org/10.1192/bjp.185.2.127

Chua, S. E., Cheung, V., McAlonan, G. M., Cheung, C., Wong, J. W. S., Cheung, E. P. T.,

Chan, M. T. Y., Wong, T. K. W., Choy, K. M., Chu, C. M., Lee, P. W. H., & Tsang, K. W. T. (2004). Stress and psychological impact on SARS patients during the outbreak. *The Canadian Journal of Psychiatry*, *49*(6), 385-390. https://doi.org/10.1177/070674370404900607

Chung, G., Lanier, P., & Wong, P. Y. J. (2022). Mediating effects of parental stress on harsh

parenting and parent-child relationship during coronavirus (COVID-19) pandemic in Singapore. *Journal of Family Violence*, *37,* 801-812. https://doi.org/10.1007/s10896-020-00200-1 *

Cipolotti, L., Chan, E., Murphy, P., van Harskamp, N., & Foley, J. A. (2021). Factors

contributing to the distress, concerns, and needs of UK Neuroscience health care workers during the COVID‐19 pandemic. *Psychology and Psychotherapy: Theory, Research and Practice*, *94*(S2), 536-543. https://doi.org/10.1111/papt.12298 *

Çolakoğlu, M. K., Özgün, Y. M., Pişkin, E., Bostancı, E. B., & Özmen, M. M. (2020). The

attitude of Turkish general surgeons during the COVID-19 pandemic: Results of" general surgery COVID-19 pandemic attitude survey". *Turkish Journal of Surgery*, *36*(2), 137-146. https://doi.org/10.5578/turkjsurg.4809

Corbett, G. A., Milne, S. J., Mohan, S., Reagu, S., Farrell, T., Lindow, S. W., Hehir, M. P., &

O’Connell, M. P. (2020). Anxiety and depression scores in maternity healthcare workers during the Covid-19 pandemic. *International Journal of Gynaecology and Obstetrics: The Official Organ of the International Federation of Gynaecology and Obstetrics*, *151*(2), 297–298. https://doi.org/10.1002/ijgo.13279

Cotrin, P., Moura, W., Gambardela-Tkacz, C. M., Pelloso, F. C., dos Santos, L., de Barros

Carvalho, M. D., Pelloso, S. M., & Freitas, K. M. S. (2020). Healthcare workers in Brazil during the COVID-19 pandemic: A cross-sectional online survey. *INQUIRY: The Journal of Health Care Organization, Provision, and Financing*, *57*, 1-11. https://doi.org/10.1177/0046958020963711

Dabholkar, Y. G., Sagane, B. A., Dabholkar, T. Y., & Divity, S. (2020). COVID19 infection in

health care professionals: Risks, work-safety and psychological issues. *Indian Journal of Otolaryngology and Head & Neck Surgery*, *72*(4), 468-473. https://doi.org/10.1007/s12070-020-01928-4

Dickinson, J. A., Bani-Adam, G., Williamson, T., Berzins, S., Pearce, C., Ricketson, L., &

Medd, E. (2013). Alberta family physicians’ willingness to work during an influenza pandemic: A cross-sectional study. *Asia Pacific Family Medicine*, *12*(3), 1-7. https://doi.org/10.1186/1447-056X-12-3

Dinibutun, S. R. (2020). Factors associated with burnout among physicians: An evaluation

during a period of COVID-19 pandemic. *Journal of Healthcare Leadership*, *12*, 85-94. http://doi.org/10.2147/JHL.S270440

Doshi, D., Karunakar, P., Sukhabogi, J. R., Prasanna, J. S., & Mahajan, S. V. (2021). Assessing

coronavirus fear in Indian population using the fear of COVID-19 scale. *International Journal of Mental Health and Addiction*, *19*, 2383-2391. https://doi.org/10.1007/s11469-020-00332-x *

Dugani, S. B., Geyer, H. L., Maniaci, M. J., Fischer, K. M., Croghan, I. T., & Burton, M. C.

(2021). Psychological wellness of internal medicine hospitalists during the COVID-19 pandemic. *Hospital Practice*, *49*(1), 47-55. https://doi.org/10.1080/21548331.2020.1832792 *

Elbay, R. Y., Kurtulmuş, A., Arpacıoğlu, S., & Karadere, E. (2020). Depression, anxiety, stress

levels of physicians and associated factors in Covid-19 pandemics. *Psychiatry Research*, *290*, 1-5. https://doi.org/10.1016/j.psychres.2020.113130

El-Zoghby, S. M., Soltan, E. M., & Salama, H. M. (2020). Impact of the COVID-19 pandemic

on mental health and social support among adult Egyptians. *Journal of Community Health*, *45*, 689-695. https://doi.org/10.1007/s10900-020-00853-5

Fang, X., Zhang, J., Teng, C., Zhao, K., Su, K. -P., Wang, Z., Tang, W., & Zhang, C. (2020).

Depressive symptoms in the front-line non-medical workers during the COVID-19 outbreak in Wuhan. *Journal of Affective Disorders*, *276*, 441-445. https://doi.org/10.1016/j.jad.2020.06.078

Fargen, K. M., Leslie-Mazwi, T. M., Klucznik, R. P., Wolfe, S. Q., Brown, P., Ansari, S. A.,

Dabus, G., Spiotta, A. M., Mokin, M., Hassan, A. E., Liebeskind, D., Welch, B. G., Siddiqui, A. H., & Hirsch, J. A. (2020). The professional and personal impact of the coronavirus pandemic on US neurointerventional practices: A nationwide survey. *Journal of Neurointerventional Surgery*, *12*(10), 927-931. http://dx.doi.org/10.1136/neurintsurg-2020-016513

Feng, Z., Xu, L., Cheng, P., Zhang, L., Li, L. -J., & Li, W. -H. (2020). The psychological impact

of COVID-19 on the families of first-line rescuers. *Indian Journal of Psychiatry*, *62*(Supplementary 3), S438-S444. http://dx.doi.org/10.4103/psychiatry.IndianJPsychiatry_1057_20

Fitzpatrick, K. M., Harris, C., & Drawve, G. (2020). Fear of COVID-19 and the mental health

consequences in America. *Psychological Trauma: Theory, Research, Practice, and Policy*, *12*(S1), S17-S21. http://dx.doi.org/10.1037/tra0000924

Foley, S. J., O’Loughlin, A., & Creedon, J. (2020). Early experiences of radiographers in Ireland

during the COVID-19 crisis. *Insights into Imaging*, *11*(104), 1-8. https://doi.org/10.1186/s13244-020-00910-6

Gan, X., Shi, Z., Chair, S. Y., Cao, X., & Wang, Q. (2020). Willingness of Chinese nurses to

practice in Hubei combating the coronavirus disease 2019 epidemic: A cross‐sectional study. *Journal of Advanced Nursing*, *76*(8), 2137-2150. https://doi.org/10.1111/jan.14434

Gassman-Pines, A., Ananat, E. O., & Fitz-Henley, J. (2020). COVID-19 and parent-child

psychological well-being. *Pediatrics*, *146*(4), 1-9. https://doi.org/10.1542/peds.2020-007294

Gershon, R. R., Magda, L. A., Qureshi, K. A., Riley, H. E., Scanlon, E., Carney, M. T., Richards,

R. J., & Sherman, M. F. (2010). Factors associated with the ability and willingness of essential workers to report to duty during a pandemic. *Journal of Occupational and Environmental Medicine*, *52*(10), 995-1003. https://doi.org/10.1097/JOM.0b013e3181f43872

Goh, S. S. N., & Chia, M. Y. C. (2020). Anxiety and morale in front-line healthcare workers

during the coronavirus disease 2019 (COVID-19) outbreak at the National Screening Centre in Singapore. *Annals of the Academy of Medicine, Singapore*, *49*(4), 259–262.

Gupta, S., Prasad, A. S., Dixit, P. K., Padmakumari, P., Gupta, S., & Abhisheka, K. (2021).

Survey of prevalence of anxiety and depressive symptoms among 1124 healthcare workers during the coronavirus disease 2019 pandemic across India. *Medical Journal Armed Forces India*, *77* (Supplementary 2), S404-S412. https://doi.org/10.1016/j.mjafi.2020.07.006 *

Han, L., Wong, F. K. Y., She, D. L. M., Li, S. Y., Yang, Y. F., Jiang, M. Y., Ruan, Y., Su, Q.,

Ma, Y., & Chung, L. Y. F. (2020). Anxiety and depression of nurses in a north west province in China during the period of novel coronavirus pneumonia outbreak. *Journal of Nursing Scholarship*, *52*(5), 564-573. http://dx.doi.org/10.1111/jnu.12590

Hasan, S. R., Hamid, Z., Jawaid, M. T., & Ali, R. K. (2020). Anxiety among doctors during

COVID-19 pandemic in secondary and tertiary care hospitals. *Pakistan Journal of Medical Sciences*, *36*(6), 1360-1365. https://doi.org/10.12669/pjms.36.6.3113

Hashim, L., Khan, H. R., Ullah, I., Khalid, M., Almas, T., Zaidi, S. M. J., Ehtesham, M., Niaz,

M. A., Akbar, A., & Haadi, A. (2020). Physician preparedness in response to the coronavirus disease 2019 pandemic: A cross-sectional study from a developing country. *Cureus*, *12*(9), 1-9. https://doi.org/10.7759/cureus.10383

Havlioğlu, S., & Demir, H. A. (2020). Determining the anxiety levels of emergency service

employees’ working during the COVID-19 pandemic. *Journal of Harran University Medical Faculty*, *17*(2), 251-255. https://doi.org/10.35440/hutfd.752467

Ho, S. M. Y., Kwong-Lo, R. S. Y., Mak, C. W. Y., & Wong, J. S. (2005). Fear of severe acute

respiratory syndrome (SARS) among health care workers. *Journal of Consulting and Clinical Psychology*, *73*(2), 344-349. https://doi.org/10.1037/0022-006X.73.2.344

Honey, M., & Wang, W. Y. Q. (2013). New Zealand nurses perceptions of caring for patients

with influenza A (H1N1). *Nursing in Critical Care*, *18*(2), 63-69. https://doi.org/10.1111/j.1478-5153.2012.00520.x

Hu, J., He, W., & Zhou, K. (2020). The mind, the heart, and the leader in times of crisis: How

and when COVID-19-triggered mortality salience relates to state anxiety, job engagement, and prosocial behavior. *Journal of Applied Psychology*, *105*(11), 1218-1233. http://dx.doi.org/10.1037/apl0000620

Huarcaya-Victoria, J., Villarreal-Zegarra, D., Podestà, A., & Luna-Cuadros, M. A. (2022).

Psychometric properties of a Spanish version of the fear of COVID-19 scale in general population of Lima, Peru. *International Journal of Mental Health and Addiction, 20*, 249-262. https://doi.org/10.1007/s11469-020-00354-5 *

Hurst, H., Ramsey, S., Jenkins, K., & Ormandy, P. (2020). UK survey of renal unit practices and

experiences of the COVID-19 pandemic. *Journal of Kidney Care*, *5*(5), 220-224. https://doi.org/10.12968/jokc.2020.5.5.220

Hwang, H., Hur, W. -M., & Shin, Y. (2021). Emotional exhaustion among the South Korean

workforce before and after COVID‐19. *Psychology and Psychotherapy: Theory, Research and Practice*, *94*(2), 371-381. https://doi.org/10.1111/papt.12309 *

Izakova, L., Breznoscakova, D., Jandova, K., Valkucakova, V., Bezakova, G., & Suvada, J.

(2020). What mental health experts in Slovakia are learning from COVID-19 pandemic? *Indian Journal of Psychiatry*, *62*(Supplementary 3), S459–S466. https://doi.org/10.4103/psychiatry.IndianJPsychiatry_758_20

Jafri, L., Ahmed, S., & Siddiqui, I. (2020). Impact of COVID-19 on laboratory professionals-A

descriptive cross sectional survey at a clinical chemistry laboratory in a developing country. *Annals of Medicine and Surgery*, *57*, 70-75. https://doi.org/[10.1016/j.amsu.2020.07.022](https://doi.org/10.1016/j.amsu.2020.07.022)

Jung, H., Jung, S. Y., Lee, M. H., & Kim, M. S. (2020). Assessing the presence of post-traumatic

stress and turnover intention among nurses post–Middle East respiratory syndrome outbreak: The importance of supervisor support. *Workplace Health & Safety*, *68*(7), 337-345. https://doi.org/10.1177/2165079919897693

Kang, L., Ma, S., Chen, M., Yang, J., Wang, Y., Li, R., Yao, L., Bai, H., Cai, Z., Yang, B. X.,

Hu, S., Zhang, K., Wang, G., Ma, C., & Liu, Z. (2020). Impact on mental health and perceptions of psychological care among medical and nursing staff in Wuhan during the 2019 novel coronavirus disease outbreak: A cross-sectional study. *Brain, Behavior, and Immunity*, *87*, 11-17. https://doi.org/10.1016/j.bbi.2020.03.028

Kelly, J. D., Hoff, N. A., Spencer, D. A., Musene, K., Bramble, M. S., McIlwain, D., Okitundu,

D., Porco, T. C., Rutherford, G. W., Glymour, M. M., Bjornson, Z., Mukadi, P., Okitolonda-Wemakoy, E., Nolan, G. P., Muyembe-Tamfum, J. J., & Rimoin, A. W. (2019). Neurological, cognitive, and psychological findings among survivors of Ebola virus disease from the 1995 Ebola outbreak in Kikwit, Democratic Republic of Congo: a cross-sectional study. *Clinical Infectious Diseases*, *68*(8), 1388-1393. https://doi.org/10.1093/cid/ciy677

Khalid, I., Khalid, T. J., Qabajah, M. R., Barnard, A. G., & Qushmaq, I. A. (2016). Healthcare

workers emotions, perceived stressors and coping strategies during a MERS-CoV outbreak. *Clinical Medicine & Research*, *14*(1), 7-14. https://doi.org/10.3121/cmr.2016.1303

Khasne, R. W., Dhakulkar, B. S., Mahajan, H. C., & Kulkarni, A. P. (2020). Burnout among

healthcare workers during COVID-19 pandemic in India: Results of a questionnaire-based survey. *Indian Journal of Critical Care Medicine: Peer-reviewed, Official Publication of Indian Society of Critical Care Medicine*, *24*(8), 664-671. https://doi.org/10.5005/jp-journals-10071-23518

Ko, N. -Y., Feng, M. -C., Chiu, D. -Y., Wu, M. -H., Feng, J. -Y., & Pan, S. -M. (2004). Applying

theory of planned behavior to predict nurses' intention and volunteering to care for SARS patients in southern Taiwan. *The Kaohsiung Journal of Medical Sciences*, *20*(8), 389-398. https://doi.org/10.1016/S1607-551X(09)70175-5

Koh, D., Lim, M. K., Chia, S. E., Ko, S. M., Qian, F., Ng, V., Tan, B. H., Wong, K. S., Chew,

W. M., Tang, H. K., Ng, W., Muttakin, Z., Emmanuel, S., Fong, N. P., Koh, G., Kwa, C. T., Tan, K. B. -C., & Fones, C. (2005). Risk perception and impact of severe acute respiratory syndrome (SARS) on work and personal lives of healthcare workers in Singapore what can we learn? *Medical Care*, *43*(7), 676-682.

Koksal, E., Dost, B., Terzi, Ö., Ustun, Y. B., Özdin, S., & Bilgin, S. (2020). Evaluation of

depression and anxiety levels and related factors among operating theater workers during the novel coronavirus (COVID-19) pandemic. *Journal of PeriAnesthesia Nursing*, *35*(5), 472-477. https://doi.org/10.1016/j.jopan.2020.06.017

Kramer, V., Papazova, I., Thoma, A., Kunz, M., Falkai, P., Schneider-Axmann, T., Hierundar,

A., Wagner, E., & Hasan, A. (2021). Subjective burden and perspectives of German healthcare workers during the COVID-19 pandemic. *European Archives of Psychiatry and Clinical Neuroscience*, *271*, 271-281. https://doi.org/10.1007/s00406-020-01183-2 *

Kumar, J., Katto, M. S., Siddiqui, A. A., Sahito, B., Ahmed, B., Jamil, M., & Ali, M. (2020).

Predictive factors associated with fear faced by healthcare workers during COVID-19 pandemic: A questionnaire-based study. *Cureus*, *12*(8), 1-6. https://doi.org/10.7759/cureus.9741

Labrague, L. J., & De los Santos, J. A. A. (2020). COVID‐19 anxiety among front‐line nurses:

Predictive role of organisational support, personal resilience and social support. *Journal of Nursing Management*, *28*(7), 1653-1661. https://doi.org/10.1111/jonm.13121

Lai, J., Ma, S., Wang, Y., Cai, Z., Hu, J., Wei, N., Wu, J., Du, H., Chen, T., Li, R., Tan, H.,

Kang, L., Yao, L., Huang, M., Wang, H., Wang, G., Liu, Z., & Hu, S. (2020). Factors associated with mental health outcomes among health care workers exposed to coronavirus disease 2019. *JAMA Network Open*, *3*(3), 1-12. https://doi.org/10.1001/jamanetworkopen.2020.3976

Lam, S. C., Arora, T., Grey, I., Suen, L. K. P., Huang, E. Y. -Z., Li, D., & Lam, K. B. H. (2020).

Perceived risk and protection from infection and depressive symptoms among healthcare workers in mainland China and Hong Kong during COVID-19. *Frontiers in Psychiatry*, *11*, 1-7. https://doi.org/10.3389/fpsyt.2020.00686

Lancee, W. J., Maunder, R. G., & Goldbloom, D. S. (2008). Prevalence of psychiatric disorders

among Toronto hospital workers one to two years after the SARS outbreak. *Psychiatric Services*, *59*(1), 91-95. https://doi.org/10.1176/ps.2008.59.1.91

Lange, M., Joo, S., Couette, P. -A., de Jaegher, S., Joly, F., & Humbert, X. (2020). Impact on

mental health of the COVID-19 outbreak among community pharmacists during the sanitary lockdown period. *Annales Pharmaceutiques Francaises*, 78(6), 459-463. https://doi.org/10.1016/j.pharma.2020.09.002

Lee, A. M., Wong, J. G., McAlonan, G. M., Cheung, V., Cheung, C., Sham, P. C., Chu, C. -M.,

Wong, P. -C., Tsang, K. W. T., & Chua, S. E. (2007). Stress and psychological distress among SARS survivors 1 year after the outbreak. *The Canadian Journal of Psychiatry*, *52*(4), 233-240. https://doi.org/10.1016/10.1177/070674370705200405

Lee, S. -H., Juang, Y. -Y., Su, Y. -J., Lee, H. -L., Lin, Y. -H., & Chao, C. -C. (2005). Facing

SARS: psychological impacts on SARS team nurses and psychiatric services in a Taiwan general hospital. *General Hospital Psychiatry*, *27*(5), 352-358. https://doi.org/10.1016/j.genhosppsych.2005.04.007

Lee, S. M., Kang, W. S., Cho, A. R., Kim, T., & Park, J. K. (2018). Psychological impact of the

2015 MERS outbreak on hospital workers and quarantined hemodialysis patients. *Comprehensive Psychiatry*, *87*, 123-127. https://doi.org/10.1016/j.comppsych.2018.10.003

Li, Q., Chen, J., Xu, G., Zhao, J., Yu, X., Wang, S., Liu, L., & Liu, F. (2020). The psychological

health status of healthcare workers during the COVID-19 outbreak: A cross-sectional survey study in Guangdong, China. *Frontiers in Public Health*, *8*, 1-9. https://doi.org/10.3389/fpubh.2020.562885

Li, T., Feng, J., Qing, P., Fan, X., Liu, W., Li, M., & Wang, M. (2014). Attitudes, practices and

information needs regarding novel influenza A (H7N9) among employees of food production and operation in Guangzhou, Southern China: A cross-sectional study. *BMC Infectious Diseases*, *14*, 1-14. https://doi.org/10.1186/1471-2334-14-4

Li, W., Frank, E., Zhao, Z., Chen, L., Wang, Z., Burmeister, M., & Sen, S. (2020). Mental

health of young physicians in China during the novel coronavirus disease 2019 outbreak. *JAMA Network Open*, *3*(6), 1-4. https://doi.org/10.1001/jamanetworkopen.2020.10705

Liu, X., Kakade, M., Fuller, C. J., Fan, B., Fang, Y., Kong, J., Guan, Z., & Wu, P. (2012).

Depression after exposure to stressful events: Lessons learned from the severe acute respiratory syndrome epidemic. *Comprehensive Psychiatry*, *53*(1), 15-23. https://doi.org/10.1016/j.comppsych.2011.02.003

Liu, Y., Wang, L., Chen, L., Zhang, X., Bao, L., & Shi, Y. (2020). Mental health status of

paediatric medical workers in China during the COVID-19 outbreak. *Frontiers in Psychiatry*, *11*, 1-7. https://doi.org/10.3389/fpsyt.2020.00702

Lu, Y. -C., Shu, B. -C., Chang, Y. -Y., & Lung, F. -W. (2006). The mental health of hospital

workers dealing with severe acute respiratory syndrome. *Psychotherapy and Psychosomatics*, *75*(6), 370-375. https://doi.org/10.1159/000095443

Lung, F. -W., Lu, Y. -C., Chang, Y. -Y., & Shu, B. -C. (2009). Mental symptoms in different

health professionals during the SARS attack: a follow-up study. *Psychiatric Quarterly*, *80*, 107-116. https://doi.org/10.1007/s11126-009-9095-5

Maciaszek, J., Ciulkowicz, M., Misiak, B., Szczesniak, D., Luc, D., Wieczorek, T., Fila-Witecka,

K., Gawlowski, P., & Rymaszewska, J. (2020). Mental health of medical and non-medical professionals during the peak of the COVID-19 pandemic: A cross-sectional nationwide study. *Journal of Clinical Medicine*, *9*(8), 1-11. https://doi.org/10.3390/jcm9082527

Majumdar, P., Biswas, A., & Sahu, S. (2020). COVID-19 pandemic and lockdown: cause of

sleep disruption, depression, somatic pain, and increased screen exposure of office workers and students of India. *Chronobiology International*, *37*(8), 1191-1200. https://doi.org/10.1080/07420528.2020.1786107

Maraqa, B., Nazzal, Z., & Zink, T. (2020). Palestinian health care workers’ stress and stressors

during COVID-19 pandemic: A cross-sectional study. *Journal of Primary Care & Community Health*, *11*, 1-7. https://doi.org/10.1177/2150132720955026

Matsuishi, K., Kawazoe, A., Imai, H., Ito, A., Mouri, K., Kitamura, N., Miyake, K., Mino, K.,

Isobe, M., Takamiya, S., Hitokoto, H., & Mita, T. (2012). Psychological impact of the pandemic (H1N1) 2009 on general hospital workers in Kobe. *Psychiatry and Clinical Neurosciences*, *66*(4), 353-360. https://doi.org/10.1111/j.1440-1819.2012.02336.x

Matsuo, T., Kobayashi, D., Taki, F., Sakamoto, F., Uehara, Y., Mori, N., & Fukui, T. (2020).

Prevalence of health care worker burnout during the coronavirus disease 2019 (COVID-19) pandemic in Japan. *JAMA Network Open*, *3*(8), 1-4. https://doi.org/10.1001/jamanetworkopen.2020.17271

Maunder, R. G., Lancee, W. J., Balderson, K. E., Bennett, J. P., Borgundvaag, B., Evans, S.,

Fernandes, C. M. B., Goldbloom, D. S., Gupta, M., Hunter, J. J., Hall, L. M., Nagle, L. M., Pain, C., Peczeniuk, S. S., Raymond, G., Read, N., Rourke, S. B., Steinberg, R. J., Stewart, T. E., VanDeVelde-Coke, S., Veldhorst, G. G., & Wasylenki, D. A. (2006). Long-term psychological and occupational effects of providing hospital healthcare during SARS outbreak. *Emerging Infectious Diseases*, *12*(12), 1924-1932. https://doi.org/10.3201/eid1212.060584

McAlonan, G. M., Lee, A. M., Cheung, V., Cheung, C., Tsang, K. W. T., Sham, P. C., Chua, S.

E., & Wong, J. G. W. S. (2007). Immediate and sustained psychological impact of an emerging infectious disease outbreak on health care workers. *The Canadian Journal of Psychiatry*, *52*(4), 241-247. https://doi.org/10.1177/070674370705200406

Mihashi, M., Otsubo, Y., Yinjuan, X., Nagatomi, K., Hoshiko, M., & Ishitake, T. (2009).

Predictive factors of psychological disorder development during recovery following SARS outbreak. *Health Psychology*, *28*(1), 91-100. https://doi.org/10.1037/a0013674

Miller, J. J., Niu, C., & Moody, S. (2020). Child welfare workers and peritraumatic distress: The

impact of COVID-19. *Children and Youth Services Review*, *119*, 1-7. https://doi.org/10.1016/j.childyouth.2020.105508

Mimoun, E., Ben Ari, A., & Margalit, D. (2020). Psychological aspects of employment

instability during the COVID-19 pandemic. *Psychological Trauma: Theory, Research, Practice, and Policy*, *12*(S1), S183-S185. http://dx.doi.org/10.1037/tra0000769

Mo, Y., Deng, L., Zhang, L., Lang, Q., Liao, C., Wang, N., Qin, M., & Huang, H. (2020). Work

stress among Chinese nurses to support Wuhan in fighting against COVID‐19 epidemic. *Journal of Nursing Management*, *28*(5), 1-8. https://doi.org/10.1111/jonm.13014

Moldofsky, H., & Patcai, J. (2011). Chronic widespread musculoskeletal pain, fatigue,

depression and disordered sleep in chronic post-SARS syndrome; A case-controlled study. *BMC Neurology*, *11*, 1-7. https://doi.org/10.1186/1471-2377-11-37

Moorthy, A., & Sankar, T. K. (2020). Emerging public health challenge in UK: Perception and

belief on increased COVID19 death among BAME healthcare workers. *Journal of Public Health*, *42*(3), 486-492. https://doi.org/10.1093/pubmed/fdaa096

Mrklas, K., Shalaby, R., Hrabok, M., Gusnowski, A., Vuong, W., Surood, S., Urichuk, L., Li, D.,

Li, X. -M., Greenshaw, A. J., & Agyapong, V. I. O. (2020). Prevalence of perceived stress, anxiety, depression, and obsessive-compulsive symptoms in health care workers and other workers in Alberta during the COVID-19 pandemic: Cross-sectional survey. *JMIR Mental Health*, *7*(9), 1-14. https://doi.org/10.2196/22408

Mulu, G. B., Kebede, W. M., Worku, S. A., Mittiku, Y. M., & Ayelign, B. (2020). Preparedness

and responses of healthcare providers to combat the spread of COVID-19 among North Shewa Zone hospitals, Amhara, Ethiopia, 2020. *Infection and Drug Resistance, 13,* 3171-3178. http://doi.org/10.2147/IDR.S265829

Murthy, P. R., Gupta, K. V. V., & Kumar, A. A. (2020). Is anxiety a rising concern during

COVID-19 pandemic among healthcare workers? *Indian Journal of Critical Care Medicine: Peer-reviewed, Official Publication of Indian Society of Critical Care Medicine*, *24*(5), 369-370. https://doi.org/10.5005/jp-journals-10071-23434

Nakhostin-Ansari, A., Sherafati, A., Aghajani, F., Khonji, M. S., Aghajani, R., & Shahmansouri,

N. (2020). Depression and anxiety among Iranian medical students during COVID-19 pandemic. *Iranian Journal of Psychiatry*, *15*(3), 228-235. https://doi.org/10.18502/ijps.v15i3.3815

Nickell, L. A., Crighton, E. J., Tracy, C. S., Al-Enazy, H., Bolaji, Y., Hanjrah, S., Hussain, A.,

Makhlouf, S., & Upshur, R. E. G. (2004). Psychosocial effects of SARS on hospital staff: Survey of a large tertiary care institution. *CMAJ*, *170*(5), 793-798. https://doi.org/10.1053/cmaj.1031077

Nie, A., Su, X., Zhang, S., Guan, W., & Li, J. (2020). Psychological impact of COVID‐19

outbreak on frontline nurses: A cross‐sectional survey study. *Journal of Clinical Nursing*, *29*(21-22), 4217-4226. https://doi.org/10.1111/jocn.15454

Odedra, D., Chahal, B. S., & Patlas, M. N. (2020). Impact of COVID-19 on Canadian radiology

residency training programs. *Canadian Association of Radiologists’ Journal*, *71*(4), 482-489. https://doi.org/10.1177/0846537120933215

Odriozola-González, P., Planchuelo-Gómez, Á., Irurtia, M. J., & de Luis-García, R. (2020).

Psychological effects of the COVID-19 outbreak and lockdown among students and workers of a Spanish university. *Psychiatry Research*, *290*, 1-8. https://doi.org/10.1016/j.psychres.2020.113108

Ogoina, D., Oyeyemi, A. S., Ayah, O., Onabor A, A., Midia, A., Olomo, W. T., & Kunle-Olowu,

O. E. (2016). Preparation and response to the 2014 Ebola virus disease epidemic in Nigeria—the experience of a tertiary hospital in Nigeria. *PLoS One*, *11*(10), 1-14. https://doi.org/10.1371/journal.pone.0165271

Oh, N., Hong, N., Ryu, D. H., Bae, S. G., Kam, S., & Kim, K. -Y. (2017). Exploring nursing

intention, stress, and professionalism in response to infectious disease emergencies: The experience of local public hospital nurses during the 2015 MERS outbreak in South Korea. *Asian Nursing Research*, *11*(3), 230-236. https://doi.org/10.1016/j.anr.2017.08.005

Pandey, U., Corbett, G., Mohan, S., Reagu, S., Kumar, S., Farrell, T., & Lindow, S. (2021).

Anxiety, depression and behavioural changes in junior doctors and medical students associated with the coronavirus pandemic: A cross-sectional survey. *The Journal of Obstetrics and Gynecology of India*, *71*, 33-37. https://doi.org/10.1007/s13224-020-01366-w *

Park, J. -S., Lee, E. -H., Park, N. -R., & Choi, Y. H. (2018). Mental health of nurses working at a

government-designated hospital during a MERS-CoV outbreak: A cross-sectional study. *Archives of Psychiatric Nursing*, *32*(1), 2-6. http://dx.doi.org/10.1016/j.apnu.2017.09.006

Paulino, M., Dumas-Diniz, R., Brissos, S., Brites, R., Alho, L., Simões, M. R., & Silva, C. F.

(2021). COVID-19 in Portugal: Exploring the immediate psychological impact on the general population. *Psychology, Health & Medicine*, *26*(1), 44-55. https://doi.org/10.1080/13548506.2020.1808236 *

Pesel, G., Canals, M. L., Sandrin, M., & Jensen, O. (2020). Wellbeing of a selection of seafarers

in Eastern Adriatic Sea during the COVID-19 pandemic 2020. *International Maritime Health*, *71*(3), 184-190. https://doi.org/10.5603/IMH.2020.0033

Pilar, A., Gravel, S. B., Croke, J., Soliman, H., Chung, P., & Wong, R. K. S. (2021). Coronavirus

disease 2019’s (COVID-19’s) silver lining—Through the eyes of radiation oncology fellows. *Advances in Radiation Oncology*, *6*(1), 1-10. https://doi.org/10.1016/j.adro.2020.07.004 *

Priolo Filho, S. R., Goldfarb, D., Zibetti, M. R., & Aznar-Blefari, C. (2020). Brazilian child

protection professionals’ resilient behavior during the COVID-19 Pandemic. *Child Abuse & Neglect*, *110*(2), 1-8. https://doi.org/10.1016/j.chiabu.2020.104701

Que, J., Shi, L. E., Deng, J., Liu, J., Zhang, L., Wu, S., Gong, Y., Huang, W., Yuan, K., Yan, W.,

Sun, Y., Ran, M., Bao, Y., & Lu, L. (2020). Psychological impact of the COVID-19 pandemic on healthcare workers: a cross-sectional study in China. *General Psychiatry*, *33*(3), 1-12. https://doi.org/10.1136/gpsych-2020-100259

Rathore, P., Kumar, S., Choudhary, N., Sarma, R., Singh, N., Haokip, N., Bhopale, S., Pandit,

A., Ratre, B. K., & Bhatnagar, S. (2020). Concerns of health-care professionals managing COVID patients under institutional isolation during COVID-19 pandemic in India: a descriptive cross-sectional study. *Indian Journal of Palliative Care*, *26*(Suppl 1), S90-S94. https://doi.org/10.4103/IJPC.IJPC_172_20

Recchi, E., Ferragina, E., Helmeid, E., Pauly, S., Safi, M., Sauger, N., & Schradie, J. (2020). The

“eye of the hurricane” paradox: An unexpected and unequal rise of well-being during the Covid-19 lockdown in France. *Research in Social Stratification and Mobility*, *68*, 1-4. https://doi.org/10.1016/j.rssm.2020.100508

Riello, M., Purgato, M., Bove, C., MacTaggart, D., & Rusconi, E. (2020). Prevalence of

post-traumatic symptomatology and anxiety among residential nursing and care home workers following the first COVID-19 outbreak in Northern Italy. *Royal Society Open Science*, *7*(9), 1-26. http://dx.doi.org/10.1098/rsos.200880

Rodriguez, R. M., Medak, A. J., Baumann, B. M., Lim, S., Chinnock, B., Frazier, R., & Cooper,

R. J. (2020). Academic emergency medicine physicians' anxiety levels, stressors, and potential stress mitigation measures during the acceleration phase of the COVID‐19 pandemic. *Academic Emergency Medicine*, *27*(8), 700-707. https://doi.org/10.1111/acem.14065

Romero, C. S., Delgado, C., Catalá, J., Ferrer, C., Errando, C., Iftimi, A., Benito, A., de Andrés,

J., & Otero, M. (2022). COVID-19 psychological impact in 3109 healthcare workers in Spain: The PSIMCOV group. *Psychological Medicine*, *52*(1), 188-194. ​​https://doi.org/10.1017/S0033291720001671 *

Rossi, R., Socci, V., Pacitti, F., Di Lorenzo, G., Di Marco, A., Siracusano, A., & Rossi, A.

(2020). Mental health outcomes among frontline and second-line health care workers during the coronavirus disease 2019 (COVID-19) pandemic in Italy. *JAMA Network Open*, *3*(5), 1-4. https://doi.org/10.1001/jamanetworkopen.2020.10185

Ruiz‐Fernández, M. D., Ramos‐Pichardo, J. D., Ibáñez‐Masero, O., Cabrera‐Troya, J.,

Carmona‐Rega, M. I., & Ortega‐Galán, Á. M. (2020). Compassion fatigue, burnout, compassion satisfaction and perceived stress in healthcare professionals during the COVID‐19 health crisis in Spain. *Journal of Clinical Nursing*, *29*(21-22), 4321-4330. https://doi.org/10.1111/jocn.15469

Ruiz-Frutos, C., Ortega-Moreno, M., Allande-Cussó, R., Ayuso-Murillo, D., Domínguez-Salas,

S., & Gómez-Salgado, J. (2021). Sense of coherence, engagement, and work environment as precursors of psychological distress among non-health workers during the COVID-19 pandemic in Spain. *Safety Science*, *133*, 1-9. https://doi.org/10.1016/j.ssci.2020.105033 *

Ruiz-Frutos, C., Ortega-Moreno, M., Allande-Cussó, R., Domínguez-Salas, S., Dias, A., &

Gómez-Salgado, J. (2021). Health-related factors of psychological distress during the COVID-19 pandemic among non-health workers in Spain. *Safety Science*, *133*, 1-10. https://doi.org/10.1016/j.ssci.2020.104996 *

Rymarowicz, J., Stefura, T., Major, P., Szeliga, J., Wallner, G., Nowakowski, M., & Pędziwiatr,

M. (2021). General surgeons’ attitudes towards COVID-19: A national survey during the SARS-CoV-2 virus outbreak. *European Surgery*, *53*, 5-10. https://doi.org/10.1007/s10353-020-00649-w *

Sadiq, M. (2022). Policing in pandemic: Is perception of workload causing work–family conflict,

job dissatisfaction and job stress? *Journal of Public Affairs*, *22*(2), 1-8. https://doi.org/10.1002/pa.2486 *

Sandesh, R., Shahid, W., Dev, K., Mandhan, N., Shankar, P., Shaikh, A., & Rizwan, A. (2020).

Impact of COVID-19 on the mental health of healthcare professionals in Pakistan. *Cureus*, *12*(7), 1-4. https://doi.org/10.7759/cureus.8974

Sarma, R., Vig, S., Rathore, P., Pushpam, D., Mishra, S., Gupta, N., Garg, R., Kumar, V.,

Bharati, S. J., & Bhatnagar, S. (2020). Concerns of health care professionals managing non-COVID patients during the COVID-19 pandemic: A descriptive cross-sectional study. *Indian Journal of Palliative Care*, *26*(Suppl 1), S21-S26. https://doi.org/10.4103/IJPC.IJPC_155_20

Savitsky, B., Findling, Y., Ereli, A., & Hendel, T. (2020). Anxiety and coping strategies among

nursing students during the covid-19 pandemic. *Nurse Education in Practice*, *46*, 1-7. https://doi.org/10.1016/j.nepr.2020.102809

Schneider, M. B., Greif, T. R., Galsky, A. P., Gomez, D., Anderson, C., Edwards, D. S., Cherry,

A. S., & Mehari, K. (2021). Giving psychology trainees a voice during the COVID-19 pandemic: Trainee mental health, perceived safety, and support. *Training and Education in Professional Psychology*, *15*(1), 76-85. http://dx.doi.org/10.1037/tep0000343 *

Shechter, A., Diaz, F., Moise, N., Anstey, D. E., Ye, S., Agarwal, S., Birk, J. L., Brodie, D.,

Cannone, D. E., Chang, B., Claassen, J., Cornelius, T., Derby, L., Dong, M., Givens, R. C., Hochman, B., Homma, S., Kronish, I. M., Lee, S. A. J. ... & Abdalla, M. (2020). Psychological distress, coping behaviors, and preferences for support among New York healthcare workers during the COVID-19 pandemic. *General Hospital Psychiatry*, *66*, 1-8. https://doi.org/10.1016/j.genhosppsych.2020.06.007

Sheng, B., Cheng, S. K. W., Lau, K. K., Li, H. L., & Chan, E. L. Y. (2005). The effects of

disease severity, use of corticosteroids and social factors on neuropsychiatric complaints in severe acute respiratory syndrome (SARS) patients at acute and convalescent phases. *European Psychiatry*, *20*(3), 236-242. https://doi.org/10.1016/j.eurpsy.2004.06.023

Shi, L., Lu, Z. -A., Que, J. -Y., Huang, X. -L., Liu, L., Ran, M. -S., Gong, Y. -M., Yuan, K.,

Yan, W., Sun, Y. -K., Shi, J., Bao, Y. -P., & Lu, L. (2020). Prevalence of and risk factors associated with mental health symptoms among the general population in China during the coronavirus disease 2019 pandemic. *JAMA Network Open*, *3*(7), 1-16. https://doi.org/10.1001/jamanetworkopen.2020.14053

Simione, L., & Gnagnarella, C. (2020). Differences between health workers and general

population in risk perception, behaviors, and psychological distress related to COVID-19 spread in Italy. *Frontiers in Psychology*, *11*, 1-17. https://doi.org/10.3389/fpsyg.2020.02166

Sin, S. S., & Huak, C. Y. (2004). Psychological impact of the SARS outbreak on a Singaporean

rehabilitation department. *International Journal of Therapy and Rehabilitation*, *11*(9), 417-424. https://doi.org/10.12968/ijtr.2004.11.9.19589

Sorokin, M. Y., Kasyanov, E. D., Rukavishnikov, G. V., Makarevich, O. V., Neznanov, N. G.,

Morozov, P. V., Lutova, N. B., & Mazo, G. E. (2020). Stress and stigmatization in health-care workers during the COVID-19 pandemic. *Indian Journal of Psychiatry*, *62*(Suppl 3), S445-S453. https://doi.org/10.4103/psychiatry.IndianJPsychiatry_870_20

Spiller, T. R., Méan, M., Ernst, J., Sazpinar, O., Gehrke, S., Paolercio, F., Petry, H., Pfaltz, M.

C., Morina, N., Aebischer, O., Gachoud, D., von Känel, R., & Weilenmann, S. (2022). Development of health care workers' mental health during the SARS-CoV-2 pandemic in Switzerland: Two cross-sectional studies. *Psychological Medicine*, *52*(7), 1395-1398. https://doi.org/10.1017/S0033291720003128 *

Styra, R., Hawryluck, L., Robinson, S., Kasapinovic, S., Fones, C., & Gold, W. L. (2008).

Impact on health care workers employed in high-risk areas during the Toronto SARS outbreak. *Journal of Psychosomatic Research*, *64*(2), 177-183. https://doi.org/10.1016/j.jpsychores.2007.07.015

Tam, C. W. C., Pang, E. P. F., Lam, L. C. W., & Chiu, H. F. K. (2004). Severe acute respiratory

syndrome (SARS) in Hong Kong in 2003: stress and psychological impact among frontline healthcare workers. *Psychological Medicine*, *34*(7), 1197-1204. https://doi.org/10.1017/S0033291704002247

Tam, D. K. P., Lee, S., & Lee, S. S. (2007). Impact of SARS on avian influenza preparedness in

healthcare workers. *Infection*, *35*, 320-325. https://doi.org/10.1007/s15010-007-6353-z

Tan, W., Hao, F., McIntyre, R. S., Jiang, L., Jiang, X., Zhang, L., Zhao, X., Zou, Y., Hu, Y.,

Luo, X., Zhang, Z., Lai, A., Ho, R., Tran, B., Ho, C., & Tam, W. (2020). Is returning to work during the COVID-19 pandemic stressful? A study on immediate mental health status and psychoneuroimmunity prevention measures of Chinese workforce. *Brain, Behavior, and Immunity*, *87*, 1-9. https://doi.org/10.1016/j.bbi.2020.04.055

Tanoue, Y., Nomura, S., Yoneoka, D., Kawashima, T., Eguchi, A., Shi, S., Harada, N., &

Miyata, H. (2020). Mental health of family, friends, and co-workers of COVID-19 patients in Japan. *Psychiatry Research*, *291*, 1-3. https://doi.org/10.1016/j.psychres.2020.113067

Tebruegge, M., Pantazidou, A., Ritz, N., Connell, T., Bryant, P., Donath, S., & Curtis, N. (2010).

Perception, attitudes and knowledge regarding the 2009 swine‐origin influenza A (H1N1) virus pandemic among health‐care workers in Australia. *Journal of Paediatrics and Child Health*, *46*(11), 673-679. https://doi.org/10.1111/j.1440-1754.2010.01820.x

Teng, Z., Wei, Z., Qiu, Y., Tan, Y., Chen, J., Tang, H., Wu, H., Wu, R., & Huang, J. (2020).

Psychological status and fatigue of frontline staff two months after the COVID-19 pandemic outbreak in China: A cross-sectional study. *Journal of Affective Disorders*, *275*, 247-252. https://doi.org/10.1016/j.jad.2020.06.032

Tolomiczenko, G. S., Kahan, M., Ricci, M., Strathern, L., Jeney, C., Patterson, K., & Wilson, L.

(2005). SARS: Coping with the impact at a community hospital. *Journal of Advanced Nursing*, *50*(1), 101-110. https://doi.org/10.1111/j.1365-2648.2005.03366.x

Trougakos, J. P., Chawla, N., & McCarthy, J. M. (2020). Working in a pandemic: Exploring the

impact of COVID-19 health anxiety on work, family, and health outcomes. *Journal of Applied Psychology*, *105*(11), 1234-1245. http://dx.doi.org/10.1037/apl0000739

Tzeng, H. -M. (2004). Nurses’ professional care obligation and their attitudes towards SARS

infection control measures in Taiwan during and after the 2003 epidemic. *Nursing Ethics*, *11*(3), 277-289. https://doi.org/10.1191/096733004ne695oa

Urooj, U., Ansari, A., Siraj, A., Khan, S., & Tariq, H. (2020). Expectations, fears and

perceptions of doctors during Covid-19 pandemic. *Pakistan Journal of Medical Sciences*, *36*(COVID19-S4), COVID19-S37-COVID19-S42. https://doi.org/10.12669/pjms.36.COVID19-S4.2643

Uzun, N. D., Tekin, M., Sertel, E., & Tuncar, A. (2020). Psychological and social effects of

COVID-19 pandemic on obstetrics and gynecology employees. *Journal of Surgery and Medicine*, *4*(5), 355-358. https://doi.org/10.28982/josam.735384

Vagni, M., Maiorano, T., Giostra, V., & Pajardi, D. (2020). Coping with COVID-19: Emergency

stress, secondary trauma and self-efficacy in healthcare and emergency workers in Italy. *Frontiers in Psychology*, *11*, 1-12. https://doi.org/10.3389/fpsyg.2020.566912

Vaziri, H., Casper, W. J., Wayne, J. H., & Matthews, R. A. (2020). Changes to the work–family

interface during the COVID-19 pandemic: Examining predictors and implications using latent transition analysis. *Journal of Applied Psychology*, *105*(10), 1073-1087. http://dx.doi.org/10.1037/apl0000819

Verma, S., & Mishra, A. (2020). Depression, anxiety, and stress and socio-demographic

correlates among general Indian public during COVID-19. *International Journal of Social Psychiatry*, *66*(8), 756-762. https://doi.org/10.1177/0020764020934508

Verma, S., Mythily, S., Chan, Y. H., Deslypere, J. P., Teo, E. K., & Chong, S. A. (2004).

Post-SARS psychological morbidity and stigma among general practitioners and traditional Chinese medicine practitioners in Singapore. *Annals of the Academy of Medicine of Singapore,* *33*(6), 743-748.

Wang, H., Liu, Y., Hu, K., Zhang, M., Du, M., Huang, H., & Yue, X. (2020). Healthcare

workers’ stress when caring for COVID-19 patients: An altruistic perspective. *Nursing Ethics*, *27*(7), 1490-1500. https://doi.org/10.1177/0969733020934146

Wang, P. -W., Lu, W. -H., Ko, N. -Y., Chen, Y. -L., Li, D. -J., Chang, Y. -P., & Yen, C. -F.

(2020). COVID-19-related information sources and the relationship with confidence in people coping with COVID-19: Facebook survey study in Taiwan. *Journal of Medical Internet Research*, *22*(6), 1-8. https://doi.org/10.2196/20021

Wang, W., Song, W., Xia, Z., He, Y., Tang, L., Hou, J., & Lei, S. (2020). Sleep disturbance and

psychological profiles of medical staff and non-medical staff during the early outbreak of COVID-19 in Hubei Province, China. *Frontiers in Psychiatry*, *11*, 1-8. https://doi.org/10.3389/fpsyt.2020.00733

Wang, Y., Di, Y., Ye, J., & Wei, W. (2021). Study on the public psychological states and its

related factors during the outbreak of coronavirus disease 2019 (COVID-19) in some regions of China. *Psychology, Health & Medicine*, *26*(1), 13-22. https://doi.org/10.1080/13548506.2020.1746817 *

Wardell, J. D., Kempe, T., Rapinda, K. K., Single, A., Bilevicius, E., Frohlich, J. R., Hendershot,

C. S., & Keough, M. T. (2020). Drinking to cope during COVID‐19 pandemic: The role of external and internal factors in coping motive pathways to alcohol use, solitary drinking, and alcohol problems. *Alcoholism: Clinical and Experimental Research*, *44*(10), 2073-2083. https://doi.org/10.1111/acer.14425

White, R. G., & Van Der Boor, C. (2020). Impact of the COVID-19 pandemic and initial period

of lockdown on the mental health and well-being of adults in the UK. *BJPsych Open*, *6*(e90), 1-4. https://doi.org/10.1192/bjo.2020.79

Wilson, J. M., Lee, J., Fitzgerald, H. N., Oosterhoff, B., Sevi, B., & Shook, N. J. (2020). Job

insecurity and financial concern during the COVID-19 pandemic are associated with worse mental health. *Journal of Occupational and Environmental Medicine*, *62*(9), 686-691. https://doi.org/10.1097/JOM.0000000000001962

Wong, K. C., Han, X. A., Tay, K. S., Koh, S. B., & Howe, T. S. (2020). The psychological

impact on an orthopaedic outpatient setting in the early phase of the COVID-19 pandemic: A cross-sectional study. *Journal of Orthopaedic Surgery and Research*, *15*, 1-7. https://doi.org/10.1186/s13018-020-01862-9

Wong, T. W., Yau, J. K. Y., Chan, C. L. W., Kwong, R. S. Y., Ho, S. M. Y., Lau, C. C., Lau, F.

L., & Lit, C. H. (2005). The psychological impact of severe acute respiratory syndrome outbreak on healthcare workers in emergency departments and how they cope. *European Journal of Emergency Medicine*, *12*(1), 13-18.

Wu, P., Fang, Y., Guan, Z., Fan, B., Kong, J., Yao, Z., Liu, X., Fuller, C. J., Susser, E., Lu, J., &

Hoven, C. W. (2009). The psychological impact of the SARS epidemic on hospital employees in China: Exposure, risk perception, and altruistic acceptance of risk. *The Canadian Journal of Psychiatry*, *54*(5), 302-311. https://doi.org/10.1177/070674370905400504

Wu, P., Liu, X., Fang, Y., Fan, B., Fuller, C. J., Guan, Z., Yao, Z., Kong, J., Lu, J., & Litvak, I. J.

(2008). Alcohol abuse/dependence symptoms among hospital employees exposed to a SARS outbreak. *Alcohol & Alcoholism*, *43*(6), 706-712. https://doi.org/10.1093/alcalc/agn073

Wu, Y., Wang, J., Luo, C., Hu, S., Lin, X., Anderson, A. E., Bruera, E., Yang, X., Wei, S., &

Qian, Y. (2020). A comparison of burnout frequency among oncology physicians and nurses working on the frontline and usual wards during the COVID-19 epidemic in Wuhan, China. *Journal of Pain and Symptom Management*, *60*(1), e60-e65. https://doi.org/10.1016/j.jpainsymman.2020.04.008

Xiao, X., Zhu, X., Fu, S., Hu, Y., Li, X., & Xiao, J. (2020). Psychological impact of healthcare

workers in China during COVID-19 pneumonia epidemic: A multi-center cross-sectional survey investigation. *Journal of Affective Disorders*, *274*, 405-410. https://doi.org/10.1016/j.jad.2020.05.081

Xiaoming, X., Ming, A., Su, H., Wo, W., Jianmei, C., Qi, Z., Hua, H., Xuemei, L., Lixia, W.,

Jun, C., Lei, S., Zhen, L., Lian, D., Jing, L., Handan, Y., Haitang, Q., Xiaoting, H., Xiaorong, C., Ran, C., Qinghua, L., Xinyu, Jian, T., Jing, T., Guanghua, J., Zhiqin, H., Nkundimana, B., & Li, K. (2020). The psychological status of 8817 hospital workers during COVID-19 Epidemic: A cross-sectional study in Chongqing. *Journal of Affective Disorders*, *276*, 555-561. https://doi.org/10.1016/j.jad.2020.07.092

Xiong, H., Yi, S., & Lin, Y. (2020). The psychological status and self-efficacy of nurses during

COVID-19 outbreak: A cross-sectional survey. *INQUIRY: The Journal of Health Care Organization, Provision, and Financing*, *57*, 1-6. https://doi.org/10.1177/0046958020957114

Yang, S., Kwak, S. G., & Chang, M. C. (2021). Psychological impact of COVID‐19 on hospital

workers in nursing care hospitals. *Nursing Open*, *8*(1), 284-289. https://doi.org/10.1002/nop2.628 *

Yang, X., Zhang, Y., Li, S., & Chen, X. (2021). Risk factors for anxiety of otolaryngology

healthcare workers in Hubei province fighting coronavirus disease 2019 (COVID-19). *Social Psychiatry and Psychiatric Epidemiology*, *56*, 39-45. https://doi.org/10.1007/s00127-020-01928-3 *

Yao, Y., Tian, Y., Zhou, J., Diao, X., Cao, B., Pan, S., Di, L., Liu, Y., Chen, H., Xie, C., Yang,

Y., Li, F., Guo, Y., & Wang, S. (2020). Psychological status and influencing factors of hospital medical staff during the COVID-19 outbreak. *Frontiers in Psychology*, *11*, 1-6. https://doi.org/[10.3389/fpsyg.2020.01841](https://doi.org/10.3389/fpsyg.2020.01841)

Yin, Q., Sun, Z., Liu, T., Ni, X., Deng, X., Jia, Y., Shang, Z., Zhou, Y., & Liu, W. (2020).

Posttraumatic stress symptoms of health care workers during the corona virus disease 2019. *Clinical Psychology & Psychotherapy*, *27*(3), 384-395. https://doi.org/10.1002/cpp.2477

Ying, Y., Ruan, L., Kong, F., Zhu, B., Ji, Y., & Lou, Z. (2020). Mental health status among

family members of health care workers in Ningbo, China, during the coronavirus disease 2019 (COVID-19) outbreak: A cross-sectional study. *BMC Psychiatry*, *20*, 1-10. https://doi.org/10.1186/s12888-020-02784-w

Zandifar, A., Badrfam, R., Khonsari, N. M., Mohammadi, M. R., Asayesh, H., & Qorbani, M.

(2020). Prevalence and associated factors of posttraumatic stress symptoms and stigma among health care workers in contact with COVID-19 patients. *Iranian Journal of Psychiatry*, *15*(4), 340-350. https://doi.org/10.18502/ijps.v15i4.4303

Zhan, Y., Liu, Y., Liu, H., Li, M., Shen, Y., Gui, L., Zhang, J., Luo, Z., Tao, X., & Yu, J. (2020).

Factors associated with insomnia among Chinese front‐line nurses fighting against COVID‐19 in Wuhan: A cross‐sectional survey. *Journal of Nursing Management*, *28*(7), 1525-1535. https://doi.org/10.1111/jonm.13094

Zhang, C., Yang, L., Liu, S., Ma, S., Wang, Y., Cai, Z., Du, H., Li, R., Kang, L., Su, M., Zhang,

J., Liu, Z., & Zhang, B. (2020). Survey of insomnia and related social psychological factors among medical staff involved in the 2019 novel coronavirus disease outbreak. *Frontiers in Psychiatry*, *11*, 1-9. https://doi.org/10.3389/fpsyt.2020.00306

Zhang, S. X., Liu, J., Jahanshahi, A. A., Nawaser, K., Yousefi, A., Li, J., & Sun, S. (2020). At

the height of the storm: Healthcare staff’s health conditions and job satisfaction and their associated predictors during the epidemic peak of COVID-19. *Brain, Behavior, and Immunity, 87,* 144–146. https://doi.org/10.1016/j.bbi.2020.05.010

Zhang, S. X., Sun, S., Jahanshahi, A. A., Alvarez-Risco, A., Ibarra, V. G., Li, J., & Patty-Tito, R.

M. (2020). Developing and testing a measure of COVID-19 organizational support of healthcare workers–results from Peru, Ecuador, and Bolivia. *Psychiatry Research*, *291*, 1-4. https://doi.org/10.1016/j.psychres.2020.113174

Zhang, Y., Xie, S., Wang, P., Wang, G., Zhang, L., Cao, X., Wu, W., Bian, Y., Huang, F., Luo,

N., Luo, M., & Xiao, Q. (2020). Factors influencing mental health of medical workers during the COVID-19 outbreak. *Frontiers in Public Health*, *8*, 1-8. https://doi.org/10.3389/fpubh.2020.00491

Zhao, F., Ahmed, F., & Faraz, N. A. (2020). Caring for the caregiver during COVID-19

outbreak: Does inclusive leadership improve psychological safety and curb psychological distress? A cross-sectional study. *International Journal of Nursing Studies*, *110*, 1-11. https://doi.org/10.1016/j.ijnurstu.2020.103725

*Note*. References marked with an asterisk (*) at the end of the citation were advance online publications when we were conducting the scoping review. The references here reflect the final publication date.
