# Supplementary material for: Workers’ well-being during viral pandemics and epidemics: A scoping review
Source: Compr Psychoneuroendocrinol. 2025 Mar 4;22:100286. doi: 10.1016/j.cpnec.2025.100286 (PMC12126619; doi:10.1016/j.cpnec.2025.100286)
Supplement: Multimedia component 3 [file mmc3.docx]

**Search Strategy**

The tables below contain layman’s terms for the concepts we reviewed and the corresponding machine language from each database. “Machine language” is the subject indices of interest we gathered in the thesauri of the search engines used in the scoping review.

**MedLine via ProQuest**

| **MAIN CONCEPT: Patient** | | |
| --- | --- | --- |
| *Layman’s terms* | *Machine language* | *String* |
| Worker/employee | *MESH("Occupational Groups")* | *AB(Worker* OR employee* OR workplace* OR “at work”) OR TI(Worker* OR employee* OR workplace* OR “at work”) OR IF(Worker* OR employee* OR workplace* OR “at work”) OR MESH("Occupational Groups")* |
| **MAIN CONCEPT: Context** | | |
| *Layman’s terms* | *Machine language* | *String* |
| Pandemic | MESH("Influenza Pandemic, 1918-1919") OR MESH("Pandemics") | AB(Pandemic OR H1N1 OR Measles OR SARS OR COVID-19 OR Coronavirus OR SARS-CoV-2) OR TI(Pandemic OR H1N1 OR Measles OR SARS OR COVID-19 OR Coronavirus OR SARS-CoV-2) OR IF(Pandemic OR H1N1 OR Measles OR SARS OR COVID-19 OR Coronavirus OR SARS-CoV-2) OR MESH("Influenza Pandemic, 1918-1919") OR MESH("Pandemics") |
| Epidemic | MESH("Epidemics") | AB(Epidemic) OR TI(Epidemic) OR IF(Epidemic) OR MESH("Epidemics") |
| **MAIN CONCEPT: Outcome(s)** | | |
| *Layman’s terms* | *Machine language* | *String* |
| Wellbeing/well-being | No applicable terms found | AB(wellbeing OR well-being) OR TI(wellbeing OR well-being) OR IF(wellbeing OR well-being) |
| Wellness | No applicable terms found | AB(Wellness) OR TI(Wellness) OR IF(Wellness) |
| Mental health | MESH("Mental Health") OR MESH("Mental Health Recovery") | AB(“Mental Health”) OR TI(“Mental Health”) OR IF(“Mental Health”) OR MESH("Mental Health") OR MESH("Mental Health Recovery") |
| Anxiety | MESH("Anxiety Disorders") OR MESH("Anxiety") OR MESH("Anxiety, Separation") OR MESH("Performance Anxiety") | (AB(Anxiety) OR TI(Anxiety) OR IF(Anxiety) OR MESH("Anxiety Disorders") OR MESH("Anxiety") OR MESH("Anxiety, Separation") OR MESH("Performance Anxiety")) |
| Burnout | MESH("Burnout, Psychological") OR MESH("Burnout, Professional") OR MESH("Occupational Stress") | AB(Burnout) OR TI(Burnout) OR IF(Burnout) OR MESH("Burnout, Psychological") OR MESH("Burnout, Professional") OR MESH("Occupational Stress") |
| Depression | MESH("Depression") | AB(Depression) OR TI(Depression) OR IF(Depression) OR MESH("Depression") |
| Distress | MESH("Psychological Distress") | AB(Distress) OR TI(Distress) OR IF(Distress) OR MESH("Psychological Distress") |
| Stress | MESH("Stress, Psychological") | AB(Stress) OR TI(Stress) OR IF(Stress) OR MESH("Stress, Psychological") |
| Performance | MESH("Employee Performance Appraisal") OR MESH("Task Performance and Analysis") OR MESH("Work Performance") | AB(Performance) OR TI(Performance) OR IF(Performance) OR MESH("Employee Performance Appraisal") OR MESH("Task Performance and Analysis") OR MESH("Work Performance") |
| Productivity | MESH("Efficiency") | AB(Productivity) OR TI(Productivity) OR IF(Productivity) OR MESH("Efficiency") |
| Self-compassion | No applicable terms found | AB(Self-compassion) OR TI(Self-compassion) OR IF(Self-compassion) |
| Character strength | No applicable terms found | AB(“Character strength”) OR TI(“Character strength”) OR IF(“Character strength”) |
| Resilience | MESH("Resilience, Psychological") | AB(resilienc*) OR TI(resilienc* ) OR IF(resilienc*) OR MESH("Resilience, Psychological") |

| ***Final string*** | ***Number of results*** | ***Date of search*** |
| --- | --- | --- |
| *((AB(Worker* OR employee* OR workplace* OR "at work") OR TI(Worker* OR employee* OR workplace* OR "at work") OR IF(Worker* OR employee* OR workplace* OR "at work") OR MESH("Occupational Groups")) AND ((AB(Pandemic OR H1N1 OR Measles OR SARS OR COVID-19 OR Coronavirus OR SARS-CoV-2) OR TI(Pandemic OR H1N1 OR Measles OR SARS OR COVID-19 OR Coronavirus OR SARS-CoV-2) OR IF(Pandemic OR H1N1 OR Measles OR SARS OR COVID-19 OR Coronavirus OR SARS-CoV-2) OR MESH("Influenza Pandemic, 1918-1919") OR MESH("Pandemics")) OR (AB(Epidemic) OR TI(Epidemic) OR IF(Epidemic) OR MESH("Epidemics")) AND ((AB(wellbeing OR well-being) OR TI(wellbeing OR well-being) OR IF(wellbeing OR well-being)) OR (AB(Wellness) OR TI(Wellness) OR IF(Wellness)) OR (AB("Mental Health") OR TI("Mental Health") OR IF("Mental Health") OR MESH("Mental Health") OR MESH("Mental Health Recovery")) OR (AB(Anxiety) OR TI(Anxiety) OR IF(Anxiety) OR MESH("Anxiety Disorders") OR MESH("Anxiety") OR MESH("Anxiety, Separation") OR MESH("Performance Anxiety")) OR (AB(Burnout) OR TI(Burnout) OR IF(Burnout) OR MESH("Burnout, Psychological") OR MESH("Burnout, Professional") OR MESH("Occupational Stress")) OR (AB(Depression) OR TI(Depression) OR IF(Depression) OR MESH("Depression")) OR (AB(Distress) OR TI(Distress) OR IF(Distress) OR MESH("Psychological Distress")) OR (AB(Stress) OR TI(Stress) OR IF(Stress) OR MESH("Stress, Psychological")) OR (AB(Performance) OR TI(Performance) OR IF(Performance) OR MESH("Employee Performance Appraisal") OR MESH("Task Performance and Analysis") OR MESH("Work Performance")) OR (AB(Productivity) OR TI(Productivity) OR IF(Productivity) OR MESH("Efficiency")) OR (AB(Self-compassion) OR TI(Self-compassion) OR IF(Self-compassion)) OR (AB("Character strength") OR TI("Character strength") OR IF("Character strength")) OR (AB(resilienc*) OR TI(resilienc*) OR IF(resilienc*) OR MESH("Resilience, Psychological")))))* | *1,315* | *October 22nd, 2020* |

**PsycINFO via Proquest**

| **MAIN CONCEPT: Patient** | |  |
| --- | --- | --- |
| *Layman’s terms* | *Machine language* | *String* |
| Worker(s)/employee(s) | *MAINSUBJECT("Personnel")* | *AB(Worker* OR employee* OR workplace* OR “at work”) OR TI(Worker* OR employee* OR workplace* OR “at work”) OR IF(Worker* OR employee* OR workplace* OR “at work”) OR MAINSUBJECT("Personnel")* |
| **MAIN CONCEPT: Context** | |  |
| *Layman’s terms* | *Machine language* | *String* |
| Pandemic/epidemic | MAINSUBJECT("Pandemics") | AB(Pandemic OR H1N1 OR Measles OR SARS OR COVID-19 OR Coronavirus OR SARS-CoV-2) OR TI(Pandemic OR H1N1 OR Measles OR SARS OR COVID-19 OR Coronavirus OR SARS-CoV-2) OR IF(Pandemic OR H1N1 OR Measles OR SARS OR COVID-19 OR Coronavirus OR SARS-CoV-2) OR MAINSUBJECT("Pandemics") |
| Epidemic | MAINSUBJECT("Epidemics") | AB(Epidemic) OR TI(Epidemic) OR IF(Epidemic) OR MAINSUBJECT("Epidemics") |
| **MAIN CONCEPT: Outcome(s)** | |  |
| *Layman’s terms* | *Machine language* | *String* |
| Wellbeing/well-being | MAINSUBJECT("Well Being") | *AB(Wellbeing OR well-being) OR TI(Wellbeing OR well-being) OR IF(Wellbeing OR well-being) OR* MAINSUBJECT("Well Being") |
| Wellness | MAINSUBJECT("Health") | *AB(Wellness) OR TI(Wellness) OR IF(Wellness) OR* MAINSUBJECT("Health") |
| Mental health | MAINSUBJECT("Public Mental Health") OR MAINSUBJECT("Community Mental Health") OR MAINSUBJECT("Mental Health") | AB(“Mental Health”) OR TI(“Mental Health”) OR IF(“Mental Health”) OR MAINSUBJECT("Public Mental Health") OR MAINSUBJECT("Community Mental Health") OR MAINSUBJECT("Mental Health") |
| Anxiety | MAINSUBJECT("Separation Anxiety Disorder") OR MAINSUBJECT("Computer Anxiety") OR MAINSUBJECT("Health Anxiety") OR MAINSUBJECT("Social Anxiety") OR MAINSUBJECT("Anxiety") OR MAINSUBJECT("Death Anxiety") OR MAINSUBJECT("Anxiety Disorders") OR MAINSUBJECT("Generalized Anxiety Disorder") | AB(Anxiety) OR TI(Anxiety) OR IF(Anxiety) OR MAINSUBJECT("Separation Anxiety Disorder") OR MAINSUBJECT("Computer Anxiety") OR MAINSUBJECT("Health Anxiety") OR MAINSUBJECT("Social Anxiety") OR MAINSUBJECT("Anxiety") OR MAINSUBJECT("Death Anxiety") OR MAINSUBJECT("Anxiety Disorders") OR MAINSUBJECT("Generalized Anxiety Disorder") |
| Burnout | MAINSUBJECT("Occupational Stress") | AB(Burnout) OR TI(Burnout) OR IF(Burnout) OR MAINSUBJECT("Occupational Stress") |
| Depression | MAINSUBJECT("Reactive Depression") OR MAINSUBJECT("Treatment Resistant Depression") OR MAINSUBJECT("Late Life Depression") OR MAINSUBJECT("Major Depression") OR MAINSUBJECT("Long-term Depression (Neuronal)") OR MAINSUBJECT("Depression (Emotion)") OR MAINSUBJECT("Recurrent Depression") OR MAINSUBJECT("Atypical Depression") OR MAINSUBJECT("Sadness") | AB(Depression) OR TI(Depression) OR IF(Depression) OR MAINSUBJECT("Reactive Depression") OR MAINSUBJECT("Treatment Resistant Depression") OR MAINSUBJECT("Late Life Depression") OR MAINSUBJECT("Major Depression") OR MAINSUBJECT("Long-term Depression (Neuronal)") OR MAINSUBJECT("Depression (Emotion)") OR MAINSUBJECT("Recurrent Depression") OR MAINSUBJECT("Atypical Depression") OR MAINSUBJECT("Sadness") |
| Distress | MAINSUBJECT("Distress") | AB(Distress) OR TI(Distress) OR IF(Distress) OR MAINSUBJECT("Distress") |
| Stress | MAINSUBJECT("Minority Stress") OR MAINSUBJECT("Physiological Stress") OR MAINSUBJECT("Chronic Stress") OR MAINSUBJECT("Posttraumatic Stress Disorder") OR MAINSUBJECT("Social Stress") OR MAINSUBJECT("Stress Management") OR MAINSUBJECT("Environmental Stress") OR MAINSUBJECT("Posttraumatic Stress") OR MAINSUBJECT("Stress and Coping Measures") OR MAINSUBJECT("Stress Reactions") OR MAINSUBJECT("Psychological Stress") OR MAINSUBJECT("Distress") OR MAINSUBJECT("Stress and Trauma Related Disorders") OR MAINSUBJECT("Stress") OR MAINSUBJECT("Acute Stress Disorder") OR MAINSUBJECT("Diathesis Stress Model") OR MAINSUBJECT("Occupational Stress") | AB(Stress) OR TI(Stress) OR IF(Stress) OR MAINSUBJECT("Minority Stress") OR MAINSUBJECT("Physiological Stress") OR MAINSUBJECT("Chronic Stress") OR MAINSUBJECT("Posttraumatic Stress Disorder") OR MAINSUBJECT("Social Stress") OR MAINSUBJECT("Stress Management") OR MAINSUBJECT("Environmental Stress") OR MAINSUBJECT("Posttraumatic Stress") OR MAINSUBJECT("Stress and Coping Measures") OR MAINSUBJECT("Stress Reactions") OR MAINSUBJECT("Psychological Stress") OR MAINSUBJECT("Distress") OR MAINSUBJECT("Stress and Trauma Related Disorders") OR MAINSUBJECT("Stress") OR MAINSUBJECT("Acute Stress Disorder") OR MAINSUBJECT("Diathesis Stress Model") OR MAINSUBJECT("Occupational Stress") |
| Performance | MAINSUBJECT("Job Performance") OR MAINSUBJECT("Performance") | AB(Performance) OR TI(Performance) OR IF(Performance) OR MAINSUBJECT("Job Performance") OR MAINSUBJECT("Performance") |
| Productivity | MAINSUBJECT("Productivity") OR MAINSUBJECT("Employee Productivity") | AB(Productivity) OR TI(Productivity) OR IF(Productivity) OR MAINSUBJECT("Productivity") OR MAINSUBJECT("Employee Productivity") |
| Self-compassion | MAINSUBJECT("Self-Compassion") | AB(Self-compassion) OR TI(Self-compassion) OR IF(Self-compassion) OR MAINSUBJECT("Self-Compassion") |
| Character strength | No applicable terms found | AB(“Character strength”) OR TI(“Character strength”) OR IF(“Character strength”) |
| Resilience | MAINSUBJECT("Adaptability (Personality)") OR MAINSUBJECT("Resilience (Psychological)") OR MAINSUBJECT("Posttraumatic Growth") | AB(Resilienc*) OR TI(Resilienc*) OR IF(Resilienc*) OR MAINSUBJECT("Adaptability (Personality)") OR MAINSUBJECT("Resilience (Psychological)") OR MAINSUBJECT("Posttraumatic Growth") |

| ***Final string*** | ***Number of results*** | ***Date of search*** |
| --- | --- | --- |
| *((AB(Worker* OR employee* OR workplace* OR “at work”) OR TI(Worker* OR employee* OR workplace* OR “at work”) OR IF(Worker* OR employee* OR workplace* OR “at work”) OR MESH("Occupational Groups")) AND ((*AB(Pandemic OR H1N1 OR Measles OR SARS OR COVID-19 OR Coronavirus OR SARS-CoV-2) OR TI(Pandemic OR H1N1 OR Measles OR SARS OR COVID-19 OR Coronavirus OR SARS-CoV-2) OR IF(Pandemic OR H1N1 OR Measles OR SARS OR COVID-19 OR Coronavirus OR SARS-CoV-2) OR MAINSUBJECT("Pandemics")*) OR (*AB(Epidemic) OR TI(Epidemic) OR IF(Epidemic) OR MAINSUBJECT("Epidemics")*)) AND ((AB(Wellbeing OR well-being) OR TI(Wellbeing OR well-being) OR IF(Wellbeing OR well-being) OR* MAINSUBJECT("Well Being")*) OR (AB(Wellness) OR TI(Wellness) OR IF(Wellness) OR* MAINSUBJECT("Health")*) OR (*AB(“Mental Health”) OR TI(“Mental Health”) OR IF(“Mental Health”) OR MAINSUBJECT("Public Mental Health") OR MAINSUBJECT("Community Mental Health") OR MAINSUBJECT("Mental Health")*) OR (*AB(Anxiety) OR TI(Anxiety) OR IF(Anxiety) OR MAINSUBJECT("Separation Anxiety Disorder") OR MAINSUBJECT("Computer Anxiety") OR MAINSUBJECT("Health Anxiety") OR MAINSUBJECT("Social Anxiety") OR MAINSUBJECT("Anxiety") OR MAINSUBJECT("Death Anxiety") OR MAINSUBJECT("Anxiety Disorders") OR MAINSUBJECT("Generalized Anxiety Disorder")*) OR (*AB(Burnout) OR TI(Burnout) OR IF(Burnout) OR MAINSUBJECT("Occupational Stress")*) OR (*AB(Depression) OR TI(Depression) OR IF(Depression) OR MAINSUBJECT("Reactive Depression") OR MAINSUBJECT("Treatment Resistant Depression") OR MAINSUBJECT("Late Life Depression") OR MAINSUBJECT("Major Depression") OR MAINSUBJECT("Long-term Depression (Neuronal)") OR MAINSUBJECT("Depression (Emotion)") OR MAINSUBJECT("Recurrent Depression") OR MAINSUBJECT("Atypical Depression") OR MAINSUBJECT("Sadness")*) OR (*AB(Distress) OR TI(Distress) OR IF(Distress) OR MAINSUBJECT("Distress")*) OR (*AB(Stress) OR TI(Stress) OR IF(Stress) OR MAINSUBJECT("Minority Stress") OR MAINSUBJECT("Physiological Stress") OR MAINSUBJECT("Chronic Stress") OR MAINSUBJECT("Posttraumatic Stress Disorder") OR MAINSUBJECT("Social Stress") OR MAINSUBJECT("Stress Management") OR MAINSUBJECT("Environmental Stress") OR MAINSUBJECT("Posttraumatic Stress") OR MAINSUBJECT("Stress and Coping Measures") OR MAINSUBJECT("Stress Reactions") OR MAINSUBJECT("Psychological Stress") OR MAINSUBJECT("Distress") OR MAINSUBJECT("Stress and Trauma Related Disorders") OR MAINSUBJECT("Stress") OR MAINSUBJECT("Acute Stress Disorder") OR MAINSUBJECT("Diathesis Stress Model") OR MAINSUBJECT("Occupational Stress")*) OR (*AB(Performance) OR TI(Performance) OR IF(Performance) OR MAINSUBJECT("Job Performance") OR MAINSUBJECT("Performance")*) OR (*AB(Productivity) OR TI(Productivity) OR IF(Productivity) OR MAINSUBJECT("Productivity") OR MAINSUBJECT("Employee Productivity")*) OR (*AB(Self-compassion) OR TI(Self-compassion) OR IF(Self-compassion) OR MAINSUBJECT("Self-Compassion") *) OR (*AB(“Character strength”) OR TI(“Character strength”) OR IF(“Character strength”)*) OR (*AB(Resilienc*) OR TI(Resilienc*) OR IF(Resilienc*) OR MAINSUBJECT("Adaptability (Personality)") OR MAINSUBJECT("Resilience (Psychological)") OR MAINSUBJECT("Posttraumatic Growth")*)))* | *653* | *October 22nd, 2020* |

**CINAHL via EBSCO**

| **MAIN CONCEPT: Patient** | |  |
| --- | --- | --- |
| *Layman’s terms* | *Machine language* | *String* |
| Worker(s)/employee(s) | No applicable terms found | *TI (Worker* OR employee* OR workplace* OR “at work”) OR AB (Worker* OR employee* OR workplace* OR “at work”) OR (Worker* OR employee* OR workplace* OR “at work”)* |
| **MAIN CONCEPT: Context** | |  |
| *Layman’s terms* | *Machine language* | *String* |
| Pandemic/epidemic | *(MH "Influenza, Pandemic (H1N1) 2009") OR (MH "Influenza, Swine") OR (MH "Disease Outbreaks")* | *(MH "Influenza, Pandemic (H1N1) 2009") OR (MH "Influenza, Swine") OR (MH "Disease Outbreaks") OR TI ( pandemic OR epidemic*  OR H1N1 OR Measles OR SARS OR COVID-19 OR Coronavirus OR SARS-CoV-2*) OR AB (pandemic OR epidemic*  OR H1N1 OR Measles OR SARS OR COVID-19 OR Coronavirus OR SARS-CoV-2*) OR (Pandemic OR epidemic*  OR H1N1 OR Measles OR SARS OR COVID-19 OR Coronavirus OR SARS-CoV-2*)* |
| **MAIN CONCEPT: Outcome(s)** | |  |
| *Layman’s terms* | *Machine language* | *String* |
| Wellbeing/well-being | (MH "Psychological Well-Being") OR (MH "Well-Being (Iowa NOC)") OR (MH "Spiritual Well-Being (Iowa NOC)") OR (MH "Psychological Well-Being (Iowa NOC)") OR (MH "Quality of Life (Iowa NOC)") OR (MH "Quality of Life") | (MH "Psychological Well-Being") OR (MH "Well-Being (Iowa NOC)") OR (MH "Spiritual Well-Being (Iowa NOC)") OR (MH "Psychological Well-Being (Iowa NOC)") OR (MH "Quality of Life (Iowa NOC)") OR (MH "Quality of Life") OR *TI (wellbeing OR well-being) OR AB (wellbeing OR well-being) OR (wellbeing OR well-being)* |
| Wellness | (MH "Wellness") | (MH "Wellness") OR *TI (Wellness) OR AB (Wellness) OR (Wellness)* |
| Mental health | (MH "Mental Health") OR (MH "Research, Mental Health") | (MH "Mental Health") OR (MH "Research, Mental Health") OR *TI (*"Mental Health"*) OR AB (*"Mental Health"*) OR (*"Mental Health"*)* |
| Anxiety | (MH "Anxiety") OR (MH "Social Anxiety Disorders") OR (MH "Anxiety Disorders") OR (MH "Separation Anxiety") OR (MH "Generalized Anxiety Disorder") OR (MH "Anxiety (Saba CCC)") OR (MH "Anxiety Reduction (Iowa NIC)") OR (MH "Anxiety (NANDA)") | (MH "Anxiety") OR (MH "Social Anxiety Disorders") OR (MH "Anxiety Disorders") OR (MH "Separation Anxiety") OR (MH "Generalized Anxiety Disorder") OR (MH "Anxiety (Saba CCC)") OR (MH "Anxiety Reduction (Iowa NIC)") OR (MH "Anxiety (NANDA)") OR *TI (*Anxiety*) OR AB (*Anxiety*) OR (*Anxiety*)* |
| Burnout | (MH "Burnout, Professional") | (MH "Burnout, Professional") OR *TI (*Burnout*) OR AB (*Burnout*) OR (*Burnout*)* |
| Depression | (MH "Depression") OR (MH "Depression, Reactive") OR (MH "Bipolar Disorder") OR (MH "Seasonal Affective Disorder") OR (MH "Affective Disorders, Psychotic") | (MH "Depression") OR (MH "Depression, Reactive") OR (MH "Bipolar Disorder") OR (MH "Seasonal Affective Disorder") OR (MH "Affective Disorders, Psychotic") OR *TI (*Depression*) OR AB (*Depression*) OR (*Depression*)* |
| Distress | (MH "Spiritual Distress (Saba CCC)") OR (MH "Psychological Distress") OR (MH "Symptom Distress") OR (MH "Spiritual Distress (NANDA)") OR (MH "Anticipatory Anxiety") | (MH "Spiritual Distress (Saba CCC)") OR (MH "Psychological Distress") OR (MH "Symptom Distress") OR (MH "Spiritual Distress (NANDA)") OR (MH "Anticipatory Anxiety") OR *TI (*Distress*) OR AB (*Distress*) OR (*Distress*)* |
| Stress | (MH "Stress Disorders, Post-Traumatic") OR (MH "Stress, Occupational") OR (MH "Stress, Physiological") OR (MH "Stress, Psychological") | (MH "Stress Disorders, Post-Traumatic") OR (MH "Stress, Occupational") OR (MH "Stress, Physiological") OR (MH "Stress, Psychological") OR *TI (*Stress*) OR AB (*Stress*) OR (*Stress*)* |
| Performance | (MH "Employee Performance Appraisal") OR (MH "Task Performance and Analysis") OR (MH "Job Performance") OR (MH "Role Performance Alteration (Saba CCC)") OR (MH "Altered Role Performance (NANDA)") OR (MH "Role Performance (Iowa NOC)") | (MH "Employee Performance Appraisal") OR (MH "Task Performance and Analysis") OR (MH "Job Performance") OR (MH "Role Performance Alteration (Saba CCC)") OR (MH "Altered Role Performance (NANDA)") OR (MH "Role Performance (Iowa NOC)") OR *TI (*Performance*) OR AB (*Performance*) OR (*Performance*)* |
| Productivity | (MH "Productivity") | (MH "Productivity") OR *TI (*Productivity*) OR AB (*Productivity*) OR (*Productivity*)* |
| Self-compassion | No applicable terms found | *TI (*Self-compassion*) OR AB (*Self-compassion*) OR (*Self-compassion*)* |
| Character strength | No applicable terms found | *TI (“*Character strength*”) OR AB (“*Character strength*”) OR (“*Character strength*”)* |
| Resilience | (MH "Hardiness") | (MH "Hardiness") OR *TI (*Resilience*) OR AB (*Resilience*) OR (*Resilience*)* |

| ***Final string*** | ***Number of results*** | ***Date of search*** |
| --- | --- | --- |
| *((TI (Worker* OR employee* OR workplace* OR “at work”) OR AB (Worker* OR employee* OR workplace* OR “at work”) OR (Worker* OR employee* OR workplace* OR “at work”)) AND ((MH "Influenza, Pandemic (H1N1) 2009") OR (MH "Influenza, Swine") OR (MH "Disease Outbreaks") OR TI ( pandemic OR epidemic*  OR H1N1 OR Measles OR SARS OR COVID-19 OR Coronavirus OR SARS-CoV-2*) OR AB (pandemic OR epidemic*  OR H1N1 OR Measles OR SARS OR COVID-19 OR Coronavirus OR SARS-CoV-2*) OR (Pandemic OR epidemic*  OR H1N1 OR Measles OR SARS OR COVID-19 OR Coronavirus OR SARS-CoV-2*)) AND ((*(MH "Psychological Well-Being") OR (MH "Well-Being (Iowa NOC)") OR (MH "Spiritual Well-Being (Iowa NOC)") OR (MH "Psychological Well-Being (Iowa NOC)") OR (MH "Quality of Life (Iowa NOC)") OR (MH "Quality of Life") OR *TI (wellbeing OR well-being) OR AB (wellbeing OR well-being) OR (wellbeing OR well-being)) OR (*(MH "Wellness") OR *TI (Wellness) OR AB (Wellness) OR (Wellness)) OR (*(MH "Mental Health") OR (MH "Research, Mental Health") OR *TI (*"Mental Health"*) OR AB (*"Mental Health"*) OR (*"Mental Health"*)) OR (*(MH "Anxiety") OR (MH "Social Anxiety Disorders") OR (MH "Anxiety Disorders") OR (MH "Separation Anxiety") OR (MH "Generalized Anxiety Disorder") OR (MH "Anxiety (Saba CCC)") OR (MH "Anxiety Reduction (Iowa NIC)") OR (MH "Anxiety (NANDA)") OR *TI (*Anxiety*) OR AB (*Anxiety*) OR (*Anxiety*)) OR (*(MH "Burnout, Professional") OR *TI (*Burnout*) OR AB (*Burnout*) OR (*Burnout*)) OR (*(MH "Depression") OR (MH "Depression, Reactive") OR (MH "Bipolar Disorder") OR (MH "Seasonal Affective Disorder") OR (MH "Affective Disorders, Psychotic") OR *TI (*Depression*) OR AB (*Depression*) OR (*Depression*)) OR (*(MH "Spiritual Distress (Saba CCC)") OR (MH "Psychological Distress") OR (MH "Symptom Distress") OR (MH "Spiritual Distress (NANDA)") OR (MH "Anticipatory Anxiety") OR *TI (*Distress*) OR AB (*Distress*) OR (*Distress*)) OR (*(MH "Stress Disorders, Post-Traumatic") OR (MH "Stress, Occupational") OR (MH "Stress, Physiological") OR (MH "Stress, Psychological") OR *TI (*Stress*) OR AB (*Stress*) OR (*Stress*)) OR (*(MH "Employee Performance Appraisal") OR (MH "Task Performance and Analysis") OR (MH "Job Performance") OR (MH "Role Performance Alteration (Saba CCC)") OR (MH "Altered Role Performance (NANDA)") OR (MH "Role Performance (Iowa NOC)") OR *TI (*Performance*) OR AB (*Performance*) OR (*Performance*)) OR (*(MH "Productivity") OR *TI (*Productivity*) OR AB (*Productivity*) OR (*Productivity*)) OR (TI (*Self-compassion*) OR AB (*Self-compassion*) OR (*Self-compassion*)) OR (TI (“*Character strength*”) OR AB (“*Character strength*”) OR (“*Character strength*”)) OR (*(MH "Hardiness") OR *TI (*Resilience*) OR AB (*Resilience*) OR (*Resilience*))))* | *553* | *October 22nd, 2020* |
